# Transient cell states encode positional information to direct asymmetric growth

**DOI:** 10.64898/2026.05.21.726925

**Published:** 2026-05-22

**Authors:** Travis A. Lee, Kamonkan Gamnerdsiri, Natanella Illouz-Eliaz, Tatsuya Nobori, Joseph R. Nery, Bruce Jow, Michelle Liem, Caz O’Connor, Joseph R. Ecker

**Affiliations:** 1 Plant Biology Laboratory, The Salk Institute for Biological Studies, La Jolla, CA, USA; 2 Howard Hughes Medical Institute, The Salk Institute for Biological Studies, La Jolla, CA, USA; 3 Genomic Analysis Laboratory, The Salk Institute for Biological Studies, La Jolla, CA, USA; 4 Flow Cytometry Core Facility, The Salk Institute for Biological Studies, La Jolla, CA, USA

## Abstract

How tissues generate asymmetric growth from spatially and temporally restricted signals remains a fundamental challenge in developmental biology. We show that transient cell states encode positional information that instructs asymmetric growth in plants. Using spatial single-cell transcriptomics and multi-omics, we uncover a surprisingly complex landscape of transient cell states in the apical hook, including a previously unrecognized population in the apical hypocotyl region that integrates developmental and hormonal cues and bifurcates into opposing growth trajectories. Gene regulatory network analysis and functional perturbation establish GATA TRANSCRIPTION FACTOR 2 (GATA2) as a central regulator that promotes cell elongation through gibberellin signaling. These results reveal that diverse, spatially localized transient cell states function as regulatory hubs that convert positional information into divergent growth programs, providing a unifying framework for how dynamic cellular states shape organ morphology.

In plants and animals, the formation of organs and tissues requires precise morphometric changes in both developmental time and space^1^. Yet, the precise timing and location of the molecular mechanisms that underlie diverse tissue shapes remain incompletely understood due to the transient regulation of symmetry-breaking events^2,3^. To achieve diverse morphologies, organisms utilize a range of growth strategies, including anisotropic and differential growth, to drive directional growth conflicts that lead to local tissue deformation^4^. While much is understood regarding the role of directional auxin flow to generate local auxin maxima to drive localized growth^5^, our understanding of the precise location and activity of genes and regulatory networks involved in differential growth remains incomplete due to compounding factors that drive cell neighborhood complexity, including transient^6^ and concentration-dependent^7^ hormonal regulation.

One of the hallmark phenotypes of dark-grown (etiolated) dicot seedlings, the transient U-shaped hypocotyl apical hook structure, is necessary for survival as seedlings emerge from the environment following germination^8^, and is regulated by asymmetric auxin and ethylene activity within cells along the concave-convex axis^9^. To initiate and maintain the apical hook, concentrically arranged hypocotyl cell types transiently undergo differential growth, which results in asymmetric cell elongation along the curvature of the apical hook^10–13^. While analysis of mutants has led to mechanistic understanding of the apical hook [reviewed by Wang et al.^14^], detailed high-resolution profiling of this transient structure, that is present for only the first 120 hours of growth^15^, remains incomplete due to the compact and complex regulation of individual cells and cell neighborhoods within the apical hook, that lie at the intersection of developmental maturation, and opposing auxin and ethylene hormonal gradients.

To address these knowledge gaps, we leveraged spatial and single-nucleus transcriptomics, as well as multi-omics, to investigate apical hook development and cell-level heterogeneity in time and space at single-cell resolution. We identified novel developmental regulatory patterns that underlie cell-level heterogeneity within the apical hook, and we focus on a population of cells immediately basal to the shoot meristem in the apical hypocotyl region (AHR), which integrates developmental and hormonal regulation to asymmetrically and divergently regulate elongation in cells of the apical hook. Gene regulatory network (GRN) analysis of these AHR cells reveals a mechanism in which their development bifurcates to regulate asymmetric elongation along the convex-concave axis. Further, we identify mechanistic regulators of apical hook development, including *GATA TRANSCRIPTION FACTOR 2* (*GATA2*), which functions as a molecular fulcrum to promote cell elongation asymmetrically within AHR cells.

## Cell types and states in the apical hook

**Fig. 1.**
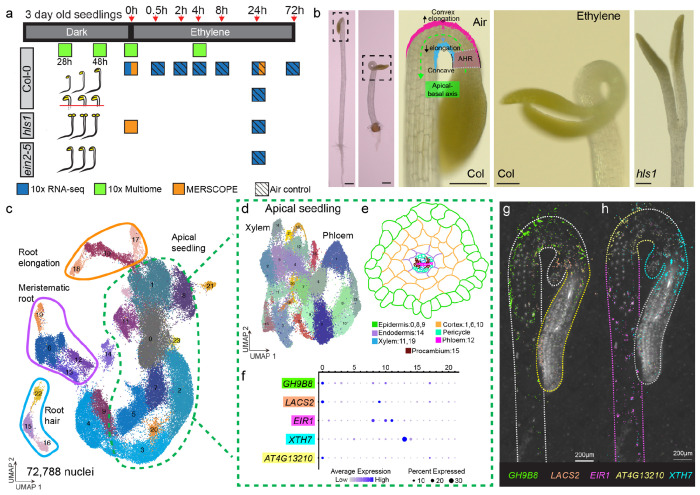
Single-cell transcriptomics resolves a diversity of cell types and states in the apical hook. **a,** Schematic of spatial and single-nucleus transcriptomics and multiomics of Arabidopsis seedlings. **b,** Representative images of etiolated and exaggerated apical hooks in air and ethylene-treated conditions (WT), and the lack of an apical hook in the *hls1* mutant. Diagram of the apical hypocotyl region (AHR) and apical-basal and convex-concave axes present within the apical hook are depicted. Scale bars, 500 μm (left) and 200 μm (right). **c,** UMAP dimensionality reduction of snRNA-seq datasets. **d,e,** Re-clustering of cells that represent the apical seedling, including the apical hook, shoot apical meristem, and cotyledons (**d**). Representation of cell types present within the hypocotyl (**e**). **f-h,** Expression of select markers identified in the apical seedling dataset (**f**). Spatial expression of the marker genes depicted in (**f**) that correspond to epidermal cells broadly (**g**), and to region-specific epidermal subpopulations (**h**). DAPI signal is depicted in gray. Scale bars, 200 μm.

To identify cells in the apical hook, we performed single-nucleus RNA sequencing (snRNA-seq) on whole etiolated seedlings over a time course of ethylene treatment (Fig. 1a,b). In parallel, We also performed snRNA-seq on dissected apical tissue of WT seedlings, in addition to whole seedlings of genetic mutants that specifically lack the apical hook structure (*hookless1*; *hls1* [*AT4G37580*]) and are insensitive to ethylene (*ethylene insensitive2-5*; *ein2-5* [*AT5G03280*]). Integration of these datasets revealed 24 clusters, many of which are annotated as discrete cell types based on the expression of canonical marker genes (Fig. 1c and Extended Data Fig. 1 and Supplementary Table 1).

As anticipated, we found that ethylene treatment induces transcriptional responses both broadly across cell types, but we also identified unique cell type-specific ethylene responses (Extended Data Fig. 2 and Supplementary Table 2 and 3), which is exemplified by our capture of ethylene-induced ectopic root hair production (clusters 15, 16, and 22; Extended Data Fig. 3)^16,17^. Importantly, we found that in the context of whole seedlings, nine clusters predominantly represent cells of the apical seedlings, providing single-cell-resolution molecular ground truth for cell-type-and state-specific expression within the apical hook.

## Transient states compose the apical hook

To investigate cell state diversity that drives asymmetric cell elongation within the apical hook, we re-clustered the cells that comprise the apical hook tissue, as defined by clusters that are enriched for the dissected apical hook tissue, and are underrepresented by *hls1* seedlings that specifically lack the apical hook structure (Fig. 1c and Extended Data Fig. 4), which expanded the nine initial clusters to 22 clusters (apical seedling, Fig. 1d). Consistent with anatomy of this tissue subset (Fig. 1e), we found that individual clusters represented cell types, cell states, and the combination therein, which may be attributed to the combinatorial activity of developmental and hormonal regulation of cells within the apical hook structure (Fig. 1d, Extended Data Fig. 4c,d, and Supplementary Table 4).

Examination of the spatial expression of markers within this dataset revealed that individual clusters broadly correspond to cell types, as well as to subpopulations or regions within individual cell types (Fig. 1g,h and Extended Data Fig. 4f). For example, we validated epidermal layer-specific markers (cluster 0) that are broadly expressed in epidermal cells of the hypocotyl and cotyledon (Fig. 1g). In contrast, we also verified markers with epidermis-specific expression that are instead restricted to defined regions of seedlings (Figs. 1f,h). These results are exemplified by the regional expression of *ETHYLENE INSENSITIVE ROOT 1* (*EIR1*; *AT5G57090*), *XYLOGLUCAN ENDOTRANSGLUCOSYLASE/ HYDROLASE 7* (*XTH7*; *AT4G37800*) (cluster 13), and a Pectin lyase-like superfamily protein (*AT4G13210*), where expression of these markers is restricted to epidermal cell populations of the basal cotyledon, basal region of the apical hook, and the straightened hypocotyl, respectively (Fig. 1h). Collectively, these results highlight the cellular complexity within the apical hook and reinforce the observed transcriptional diversity within our focused apical hook single-nucleus dataset.

## Position encoded cell states

**Fig. 2.**
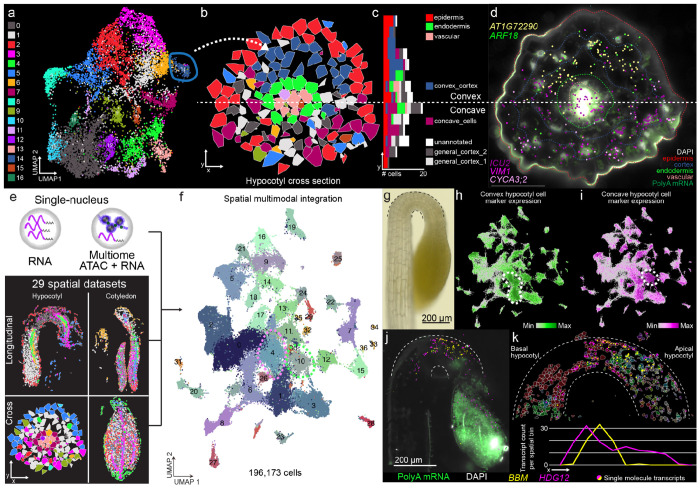
Spatial cell states integrate developmental and hormonal signals in the apical hook. **a-d,** Spatial single-cell analysis of MERFISH datasets reveals spatially distinct cortex subpopulations. Dimensionality reduction (**a**) and spatial representation of a hypocotyl cross-section (**b**) of cell-segmented MERFISH datasets. Convex cortex cells (cluster 14) are circled for emphasis. (**c**) Quantitation of cell centroids along the y-axis as depicted in (B). Spatial detection of markers corresponding to convex vs. concave cortex cells is depicted. Single-molecule transcripts are colored accordingly. Broken lines represent outlines of cell layers; DAPI and PolyA mRNA signals are false-colored white and green, respectively. Scale bar, 50 μm (**d**). **e,f,** Schematic (**e**) and dimensionality reduction and clustering (**f**) of single-nucleus and spatial datasets. Clusters 4 and 10 are circled for emphasis. **g,** Representative image of a wild-type apical hook. Scale bar, 200 μm. **h,i,** Module expression of cluster 4 (**h**) and 10 markers (**i**) corresponding to convex and concave hypocotyl cells, respectively. **j,k,** Spatial regulation of early developmental regulators in the apical hook. Single-molecule detection of *BBM* (yellow) and *HDG12* (magenta) transcripts in the apical seedling region (**j**). DAPI and PolyA mRNA signal are colored white and green, respectively. Scale bar, 200 μm. (Top) A zoomed-in region of the apical hook is depicted with cell segmentations (**k**). Spatial cells are colored according to cluster identity as depicted in (**f**). (Bottom) Quantitation of *BBM* and *HDG12* transcripts within spatial bins of the depicted apical hook region along the x-axis.

To quantitatively investigate cell states within the apical hook, we performed transcript-based cell segmentation of the 29 MERFISH datasets, followed by integration (Fig. 2a and Extended Data Figs. 5 and 6). As expected, *de novo* clustering of the spatial single-cell datasets identified clusters that correspond to cell types (Fig. 2b and Extended Data Fig. 6b,c). Interestingly, *de novo* clustering of the spatial datasets also identified clusters that correspond to cell subpopulations, such as convex cortex cells of the apical hook (cluster 14, Fig. 2a–c and Extended Data Fig. 6). Examination of markers of the convex cortex cluster revealed genes regulated by both auxin and ethylene that are concordantly spatially enriched within convex cortex cells (Fig. 2d). Of note, *AT1G72290*, a member of the small Kunitz family trypsin and protease inhibitor proteins, is known to be induced by ethylene and accumulates specifically within the convex region of the apical hook of Arabidopsis seedlings^18^, which further validates the spatial segmentation, integration, and analysis of our MERFISH datasets (Fig. 2d and Extended Data Fig. 6).

We next integrated our spatial single-cell MERFISH datasets with the droplet-based single-nucleus RNA and multiome (ATAC + RNA) datasets (Fig. 2e) to generate a spatial multimodal dataset (Fig. 2f). We found that this integrated dataset retains clusters that correspond both to cell types and cell states, including cell states that correspond to the convex and concave regions of the apical hook (Fig. 2g–i). Interestingly, we also identified and spatially validated clusters that correspond to other cell states and/or cell subtypes, as exemplified by adaxial epidermal cells and distal epidermal cells of cotyledons (Extended Data Fig. 7). From our spatial multi-modal dataset, we were able to comprehensively identify cell-type and cell-state marker genes (Supplementary Table 5), as well as their putative TF regulators inferred from clusterspecific accessible chromatin (Extended Data Fig. 7, Supplementary Table 6) and their corresponding spatial location within seedlings, which elucidated the regulators and genes involved in cell type and cell state specification.

Interestingly, we also identified several markers related to developmental progression and cell cycle regulation (Fig. 2d). Examination of the spatial expression of known early growth regulators, such as *BABY BOOM* (*BBM; AT5G17430*)^19^ and *HOMEODOMAIN GLABROUS 12* (*HDG12*; *AT1G17920*)^20^ revealed that transcripts of these genes are not uniformly distributed within cells along the curvature of the apical hook (Fig. 2j,k and Extended Data Fig. 8). Instead, we observed maximal *BBM* transcript detection within cells immediately before the apical hook midpoint, which is flanked by *HDG12* expression (Fig. 2k, bottom). This observation is consistent with the understanding that *BBM* and *HDG12* antagonistically function through shared target genes to regulate cell proliferation and/or regeneration potential^20^. Furthermore, *BBM* and *HDG* proteins, including *HDG12*, have been shown to physically interact^20^, which may explain their phased expression pattern and suggest transient, dynamic developmental regulation within cells as they migrate along the length of the apical hook. Furthermore, *HDG11* and *HDG12* are known to promote zygote asymmetric cell division and apical-basal pattern formation during embryonic development^21^, suggesting a conserved function of genes involved in asymmetric growth regulation across developmental contexts. Collectively, these results demonstrate that the spatial position of cells along the hypocotyl apical-basal axis encodes an additional layer of cell identity, in addition to cell type, including, but not limited to, targets within the BBM and HDG regulatory network.

## *GATA2* promotes asymmetric growth

**Fig. 3.**
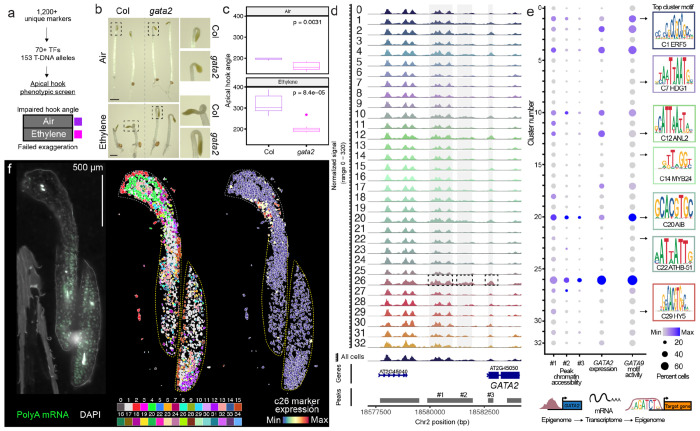
*GATA2* links transient cell states to asymmetric growth in the apical hook. **a,** Overview of the apical hook phenotypic screen performed. **b,** Representative image of WT and *gata2* etiolated seedlings when grown in control (air) or ethylene treatments for three days. Magnified images of the apical hook for the depicted seedlings of each genotype and condition are provided. **c,** Quantitation of apical hook angle in WT and *gata2* mutants in control and ethylene-treated conditions. n > 7 individual seedlings for each genotype and condition. Apical hook angle is displayed as box plots; the centre line indicates the median value, the box boundaries represent the 25th and 75th percentiles, and the whiskers extend to the minimum and maximum values within 1.5 times the interquartile range. p-values determined by unpaired t-test comparing the mutant to WT for each condition are depicted. **d-f,** *GATA2* functionally regulates the apical hook angle and its exaggeration in AHR cells. Genome track of cluster-specific chromatin accessibility within the integrated multi-modal dataset (**d**). Genomic coordinates of three peaks are specified in gray. Cluster level quantification of chromatin accessibility within the three *GATA2* promoter peaks, *GATA2* expression, and putative *GATA2* motif activity within promoter accessible chromatin of putative target genes (**e**). The top motif for select clusters is depicted to the right. Representative image (left) and depiction of spatial cells colored by cluster number (middle) and expression level of cluster 26 markers (right) are depicted (**f**). In the image, PolyA mRNA and DAPI signal are colored green and white, respectively. Scale bars, 500μm.

Based on the observed cell complexity within the seedling apical hook (Fig. 1d and 2f), we next asked whether the diverse cell subtypes and states are functionally relevant to apical hook development. Within the 154 subclusters of the focused apical hook dataset (Fig. 1d), we identified over 1,200 genes that were uniquely identified as markers for individual clusters or subclusters (Fig. 3a). To narrow our search, we focused on the 74 markers that encode transcription factors, which we phenotypically screened for defects in (1) apical hook angle in control conditions (air) and (2) apical hook exaggeration in ethylene treated conditions. From this screen, we identified two mutants of distinct transcription factor families, including *gata transcription factor 2* (*gata2*; *AT2G45050*) and *auxin response factor 6* (*arf6*; *AT1G30330*) that exhibited both (I) a reduced apical hook angle compared to WT when grown in air conditions, and (II) failed to produce an exaggerated apical hook when treated with ethylene (Fig. 3b,c and Extended Data Fig. 9), demonstrating functional roles of novel cell state markers in apical hook development. Interestingly, while mutation of *arf2* is a known suppressor of the hookless phenotype in *hls1* mutants^22^, neither *GATA2* nor *ARF6* has been previously implicated in apical hook development.

We found that expression of both *GATA2* and *ARF6* was highly enriched within a single cluster (cluster 26, Fig. 3d,e) of our spatially integrated dataset. Furthermore, we found that the promoters of both *GATA2* and *ARF6* were concordantly associated with cluster-specific chromatin accessibility within cluster 26 cells (Fig. 3d,e and Extended Data Fig. 10). Finally, using the known motifs of *GATA9* and *ARF8*, the nearest phylogenetic family members of *ARF6*^23^ and *GATA2* [subfamily 1^24^] as binding motif proxies, we found that the motif activity (motif within target gene accessible chromatin regions [ACRs]) of both *ARF8* and *GATA9* were similarly enriched within cluster 26 cells, demonstrating that both *ARF6* and *GATA2* functionally regulate apical hook development and exaggeration within these cluster 26 cells (Fig. 3e and Extended Data Fig. 10).

We found that cluster 26 cells spatially correspond to a small population of cells within the apical hypocotyl region (AHR) immediately basal to the shoot apical meristem (SAM) and meristematic region (Fig. 3f and Extended Data Fig. 11). Together, these results suggest that, rather than being cell-type-specific, cluster 26 cells specify a transient cellular state associated with developmental progression in AHR cells of the apical hook.

## Transient cell states bifurcate growth

**Fig. 4.**
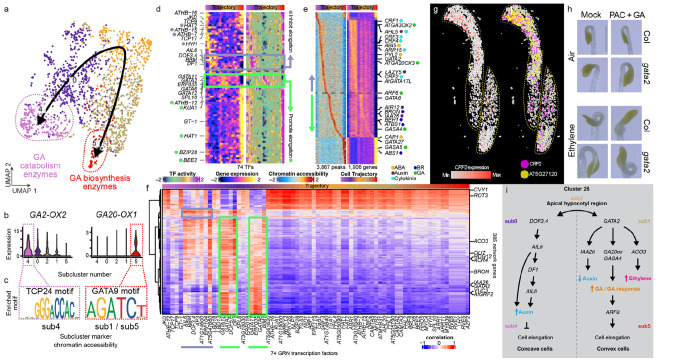
Transient cell states bifurcate growth trajectories through divergent regulatory networks **a,** Re-clustering of cluster 26 cells. Subclusters 4 and 5, associated with the expression of GA catabolism and GA biosynthesis enzymes, respectively, are circled for emphasis. The perceived cell trajectory is depicted. **b,** Violin plot of *GA2-OX2* and *GA20-OX1* expression within c26 subclusters. **c,** Promoter ACRs of subclusters 4 and 1 and 5 marker genes are enriched for *TCP24* and *GATA9* motifs, respectively. **d-f,** Gene regulatory network analysis of cluster 26 cells along the cell trajectory. (**d**) Correlation heatmap between gene expression and motif activity of inferred target genes of the 74 network TFs. Select TFs are depicted. TFs that are known to promote or inhibit cell elongation are specified in green and purple, respectively. (**e**) Correlation heatmap of promoter chromatin accessibility and gene expression of 1,906 genes regulated along the cell trajectory. Genes that are known to be regulated by ABA, auxin, BR, cytokinin, or GA are indicated accordingly. Cells along the trajectory are depicted on the x-axis. **f,** Heatmap of expression correlation between node TFs and GRN targets. Columns are ordered along the cell trajectory. **g,** Spatial expression of the GRN targets *CRF2* (magenta) and *AT5G27120* (yellow). Cell-level expression of *CRF2* (left), and single-molecule detection of both *CRF2* and *AT5G27120* are depicted (right). **h,** Images of apical hooks of Col and *gata2* mutants grown in mock or PAC + GA conditions when grown in both air (control) and ethylene-treated conditions. **i,** Proposed molecular mechanism of GRN genes that drives asymmetric cell elongation along the convex-concave axis in AHR cells.

To understand the functional role of AHR cells on apical hook regulation, we subclustered these cells, which revealed an arc-like trajectory (Fig. 4a). Interestingly, examination of subcluster markers revealed opposite and inverted expression of gibberellic acid (GA) catabolism (e.g., *GA2-OX1, GA2-OX2, GA2-OX4*, and *GAMT2*) and biosynthesis genes (e.g., *GA20-OX1, GA20-OX2*, and *GA2*)^25^ along the cell trajectory, which suggests that rather than an arc-like trajectory, divergent hormonal regulation within the transient AHR cell population instead represent a landscape-like bifurcation that leads to opposing growth trajectories to promote (subclusters 1 and 5) or suppress (subclusters 0 and 4) cell elongation among neighboring cells of a cell file(s) (Fig. 4b and Extended Data Fig. 12). These results are consistent with the understanding that both apical hook maintenance in air and apical hook enhancement under ethylene conditions depend on GA signaling^26–28^ and provide spatial context for the dynamic regulation of GA metabolism enzymes (Fig. 3f).

Leveraging our multi-modal dataset, we found that the *GATA9* motif was uniquely enriched within promoter ACRs of subclusters 1 and 5 marker genes. In contrast, promoter ACRs of subcluster 4 marker genes were instead associated with motifs of other transcription factor families, such as *TCP24* (Fig. 4c, Extended Data Fig. 12, and Supplementary Table 7). From the observed divergence of putative TF regulators along both cell trajectories (Extended Data Fig. 13), we hypothesized that distinct transcription factor networks and/or hierarchies may regulate asymmetric and differential cell elongation required for normal apical hook development^29^.

To investigate this question, we constructed a gene regulatory network (GRN, Extended Data Fig. 13a) from our multi-modal data^30^, which revealed 74 network regulators with positively correlated promoter ACR, gene expression, and motif activity of 1,906 inferred target genes along the cell trajectory (Fig. 4d–f and Extended Data Fig. 13). Interestingly, we found that the cell trajectory may instead represent a developmental bifurcation that asymmetrically regulates cell elongation along the convex-concave axis of AHR cells, with one branch suppressing cell elongation (subclusters 0 and 4) and the other promoting it (subclusters 1 and 5).

Consistent with the hypothesis that the AHR cell clustering represents a developmental bifurcation event (Fig. 4a), we found that many of the genes within the constructed GRN are known to be regulated by diverse hormones with opposing roles in cell elongation, and several of which are known regulators of apical hook development^14^ (Fig. 4d,e). For example, within the cell elongation suppression trajectory, we identified genes involved in cytokinin [e.g., *CYTOKININ RESPONSE FACTOR 1, 3, and 4* (*CRF1, 3, 4*)^31^] and abscisic acid (ABA) (e.g., *ABA INSENSITIVE 5* [*ABI5*] and *PYR1-like 2* [*PYL2*]) signaling pathways, where both ABA and cytokinin are known to generally inhibit cell elongation in hypocotyls and other developmental contexts^32–34^. Conversely, within the cell elongation trajectory we instead found genes involved in auxin (*AUXIN-INDUCED IN ROOT CULTURES 12* [*AIR12*]^35^ and *INDOLE-3-ACETIC ACID INDUCIBLE 26* [*IAA26*]), brassinosteroid (BR) (*BRONTOSAURUS* [*BRON*]^36^ and *BES1/BZR1 HOMOLOG 1* [*BEH1*]), and GA (*GAST1 PROTEIN HOMOLOG 4* [*GASA4*] and 5 [*GASA5*]) signaling pathways, where these hormones generally promote cell elongation^32,37–39^ (Fig. 4e). Our GRN also revealed an early role of *GATA2* in the cell elongation trajectory and predicted that it directly regulates several hormonally regulated genes within the constructed GRN (Fig. 4f, Extended Data Fig. 13d, and Supplementary Table 8), which may explain the reduced apical hook angle observed in *gata2* mutants (Fig. 3b).

In addition to intersecting hormonal signaling pathways, our GRN also predicts the regulation of several genes that functionally regulate cell elongation within the hypocotyl. For example, *AT HOOK MOTIF DNA-BINDING FAMILY PROTEIN 5* (*AHL5*)^40^*, HOMEOBOX-LEUCINE ZIPPER PROTEIN 4* (*HAT2*)^41^, and *HY5-HOMOLOG* (*HY5*)^42^ all functionally inhibit hypocotyl cell elongation, whereas *KUODA1* (*KUA1*)^43^, *HOMEODOMAIN-LEUCINE ZIPPER 1* (*HAT1*)^44^, and *BR ENHANCED EXPRESSION 2* (*BEE2*)^45^ all functionally promote hypocotyl elongation (Fig. 4d).

To spatially validate our GRN, we examined the spatial expression of genes predicted to be involved in the cell elongation suppression trajectory (*CRF2* and *AT5G27120*). We found that, although expression of these genes was not unique to the apical hook, we observed asymmetric transcript distribution of both *CRF2* and *AT5G27120*, with spatially enriched expression in concave apical hook cells, which are associated with reduced cell elongation (Fig. 4g and Extended Data Fig. 14). Additionally, in the *hls1* mutant that specifically lacks the apical hook structure, we found both a loss of asymmetric distribution and reduced expression of both *CRF2* and *AT5G27120*, further supporting the accurate prediction of functional cell elongation regulators in concave apical hook cells in our GRN (Fig. 4d,e and Extended Data Fig. 13 and 14).

Collectively, our GRN captures both known and novel regulators of the apical hook structure and predicts novel transcription factor network interactions that may underpin the molecular mechanisms by which *GATA2* regulates asymmetric growth in this structure. Further, our GRN sheds light on when, where, and which genes function to bifurcate cell developmental trajectories through downstream hormonal regulation by ABA, cytokinin, auxin, BR, and GA, thereby asymmetrically regulating cell elongation within the transient AHR cell population.

## *GATA2* drives asymmetric growth via GA

Finally, as our GRN predicts that *GATA2* directly regulates genes involved in GA biosynthesis and signaling to promote cell elongation (Fig. 4c–f), we hypothesized that exogenous GA application may functionally rescue the observed reduction in apical hook angle and exaggeration in *gata2* mutants (Fig. 3b). Testing this hypothesis, we found that exogenous GA was able to functionally rescue the reduced apical hook angle and apical hook exaggeration phenotypes when *gata2* mutants were grown in air and ethylene treatment, respectively (Fig. 4h).

Interestingly, while we found that exogenous GA was able to rescue the ethylene-induced apical hook exaggeration in *gata2* mutants, validating our hypothesis that *GATA2* regulates GA levels during apical hook formation, it was not able to fully rescue the hyper-exaggerated apical hook angle observed in wild-type plants (Fig. 4h and Extended Data Fig. 15), which suggests that *GATA2* may interact with other hormonal signaling pathways for spatiotemporal control of apical hook exaggeration, as predicted by our GRN (Fig. 4d–f).

Together, these results reveal a pivotal role of AHR cells as they transiently enter the apical hook, integrating diverse developmental and hormonal signals to regulate asymmetric cell elongation along the convex-concave axis of the apical hook. Furthermore, using our GRN and known genetic interactions, we constructed a molecular model in which *GATA2* functionally initiates a developmental trajectory leading to asymmetric cell elongation in cells of the apical hook (Fig. 4j).

## Discussion

In both plants and animals, precise understanding of the molecular events in time and space that result in the production of diverse tissue shapes^1^ remains challenging due to the transient and localized regulation involved in symmetry-breaking events^46–49^. While essential for survival in the wild, the apical hook is entirely dispensable under laboratory conditions and serves as a tractable model for differential growth. Using spatial and single-cell transcriptomics and multiomics, we identify a transient, spatially restricted population of cells (AHR, Fig. 3f) that positionally integrate developmental and hormonal signals to bifurcate growth programs within a local cellular neighborhood, thereby regulating differential growth in the apical hook (Fig. 4).

We find that divergent growth trajectories involve spatial partitioning of antagonistic transcription factors (Fig. 2k) that regulate cell proliferation and asymmetric growth in early embryonic development, suggesting that the described molecular mechanism of differential growth (Fig. 4j) is conserved across developmental and tissue contexts. Gene regulatory network construction reveals bifurcated transcriptional programs involving over 300 network genes, many with known roles in apical hook development and in regulating cell elongation, that drive differential growth through divergent regulation of hormone signaling pathways.

From our GRN, we identify a central function of *GATA2* in the transient and asymmetric regulation of cell elongation via GA biosynthesis and signaling genes. As GATA transcription factors are evolutionarily conserved across animal and plant species^50,51^, with functional roles in early animal development and organ specification, it is enticing to speculate that GATA members functionally regulate organ specification, initiation, and symmetry breaking across kingdoms.

While we do not identify a master regulator of differential growth in the apical hook, it is interesting to hypothesize that a combination of factors, such as the presence or absence or cell proliferation regulators, including *BBM* and *HDG12* (Fig. 2k), in combination with the presence or absence of gene regulatory network TF nodes (Fig. 4d and Extended Data Fig. 12), may provide a molecular environment suitable for TF interactions to initiate divergent transcriptional programs that drive differential growth, potentially through pioneering roles as observed for animal GATA TFs^52^. Furthermore, understanding how both intrinsic and external signals, including mechanical cues^53^ and environmental conditions^54^ integrate with the described GRN enables ample opportunity for hypothesis generation.

Collectively, these datasets highlight the complex regulatory networks that transiently govern apical hook development and hypocotyl bending and provide hundreds of novel genetic targets for the mechanistic understanding of this compact, complex apical hook structure.

## Methods

### Plant materials and growth

Wild-type Arabidopsis (Col-0) was used along with the following mutant alleles described in this study: *hls1* (CS3073), *ein2*-*5*^55^, *gata2* (SALK_106417.1 and WiscDSLox500G11), and *arf6* (SALK_104169 and SAIL_724_D08C). Arabidopsis seeds were surface-sterilized with 70% ethanol for 5 min, followed by 50% bleach and 0.1% Tween-20 for 5 min each, and washed with water for 5 min three times. Seeds were sown on 1X LS, 1% sucrose, 0.8% agar plates and incubated at 4 °C for four days in complete darkness. Following cold treatment, the plates were exposed to ambient light for 4h to synchronize germination, then placed in sealed, opaque chambers and aerated with zero-grade air for 72h at 23 °C. Following 72h of growth, the flow of zero-grade air was replaced with 30 ml/min of ethylene gas (10 ppm, Airgas) for 30 minutes, 4h, 8h, or 24h for ethylene-treated plants, and the plants were collected into liquid N_2_. Control plants were collected into liquid N_2_ following 72h or 96h of growth under continuous aeration with zero-grade air.

### Nuclei extraction and fluorescence-activated nuclei sorting (FANS)

1.5g pulverized frozen tissue was extracted in 10 mL nuclei purification buffer (NPB; 20 mM MOPS pH 7, 40 mM NaCl, 90 mM KCl, 2 mM EDTA, 0.5 mM EGTA, 0.5 mM spermidine, 0.2 mM spermine, 1X protease inhibitors (Sigma), 1:1000 SUPERase (Thermo), at 4 °C for 10 min with gentle agitation. The nuclei-containing extract was sequentially filtered through 70 μm (Fisher) and 30 μm (Miltenyi) filters at 4 °C. The filtered extract was then homogenized using a pre-chilled Dounce homogenizer with 10 loose pestle strokes followed by 10 tight pestle strokes. Nuclei were pelleted by centrifugation at 700 x g for 5 min at 4 °C and resuspended in 3 mL NPB. A discontinuous gradient of 15%/45% optiprep was prepared by diluting 60% Optiprep (Sigma) to 50% with 120 mM Tris-Cl, pH 8.0, 150 mM KCl, 30 mM MgCl_2_, and further diluted to 15% or 45% with NPB containing 1:1000 SUPERase. The nuclear pellet was resuspended in 3 mL NPB, gently layered onto the 15%/45% OptiPrep gradient, and centrifuged at 1500 × g for 15 min at 4 ° C. Following centrifugation, the nuclei were collected from the 15%/45% OptiPrep interface, washed with 10 mL of 1X PBS (Corning), and pelleted by centrifugation at 500 × g for 5 min at 4 °C. The nuclei were resuspended in 1 mL of PBS, stained with 3 μg of Hoechst 33342, placed on ice, and taken to the Flow Cytometry Core for Fluorescence-Activated Nuclei Sorting (FANS). A BD Influx sorter with a 100-μm nozzle was used to sort nuclei in 1x PBS with sheath fluid at 20 PSI; nuclei were kept at 4 °C during the purification process. Debris was excluded first by gating using forward and side scatter pulse area parameters (FSC-A and SSC-A in log scale), then exclusion of aggregates using pulse width parameters (FSC-W and SSC-W), and finally gating on Hoechst-positive nuclei. Hoechst was excited with a 405nm laser at 100mW and detected using the 460/50BP filter. The Hoechst-positive nuclei were sorted using the ‘1-drop pure’ mode directly into 1.5mL Eppendorf tubes containing 1x PBS, 0.5% BSA, and 1:1,000 SUPERase.

### Single-nucleus RNA sequencing

Following FANS, nuclei were centrifuged at 500 × g for 5 min; the supernatant was aspirated, and the nuclei were resuspended in 50 μl of PBS, BSA, and RNase inhibitors and quantified using a hemocytometer (Incyto). 10,000 nuclei, or the total nucleus suspension if fewer than 10,000 nuclei were collected, were used as input for the 10x Genomics Single Cell 3’ Gene Expression v3 reagents. Sequencing libraries were constructed following the manufacturer’s instructions and were sequenced with the Illumina NovaSeq 6000.

### Single-nucleus multiome sequencing

Nuclei were extracted as described^56^, with slight modifications. Plants were grown as described previously, and nuclei were immediately extracted from air-grown seedlings following 28h, 48h, and 72h of growth and 72 h-old seedlings following 4h ET treatment in a green room. Purified nuclei were used as input for the 10x Genomics Chromium Single Cell Multiome ATAC + Gene Expression kit. Sequencing libraries were constructed following the manufacturer’s instructions and were sequenced with the Illumina NovaSeq X.

### 1,000 target MERFISH panel design

The following criteria filtered cluster and subcluster markers identified from the apical seedling dataset: (1) more than 25 specific probes can be designed based on Vizgen’s software, and (2) the transcript per million (TPM) value is below 1000 in three-day-old seedling RNA-seq data^57^. After filtering, 600 genes representing most major clusters and subclusters were selected in addition to 400 transcription factors with expression that was specific to clusters and/or subclusters in the three-day-old seedling dataset. The panel design was balanced so that the total TPM across 1,000 transcripts was kept below 15,000 to avoid signal overcrowding.

### Tissue fixation and mounting

Plants were grown according to the previously described methods. Tissues of 3-day-old etiolated seedlings were placed into cryomolds (Sakura), which were filled with OCT and immediately frozen in an isopentane bath cooled by liquid N_2_ under a green light. Tissue blocks were acclimated to −18 °C in a pre-cooled cryostat chamber (Leica) for 1 h. Tissue blocks were trimmed until the tissue was entered, after which 10 μm sections were visually inspected until the region of interest was exposed. Sample mounting and preparation were performed according to the MERSCOPE user guide, with slight modifications. Briefly, a 10 μm section was melted and mounted onto a room-temperature MERSCOPE slide (Vizgen, 20400001), placed in a pre-chilled 60 mm Petri dish, and re-frozen by incubation in the cryostat chamber for 5 min. Subsequent steps were performed with the mounted samples in the Petri dish. The samples were then baked at 37 °C for 5 min and incubated in fixation buffer (1X PBS, 4% formaldehyde) for 15 min at RT. Samples were then washed with 1X PBS containing 1:500 RNase inhibitor (Protector RNase inhibitor, Millipore Sigma) for 5 min at RT in triplicate. Following the final PBS wash, samples were dehydrated by incubation in 70% EtOH at 4°C overnight.

### MERFISH

Tissue sections were processed following the manufacturer’s protocol. After removing 70% ethanol, the sample was incubated in the Sample Prep Wash Buffer (PN20300001) for 1 min, then in Formamide Wash Buffer (PN20300002) at 37 °C for 30 min. After removing the Formamide Wash Buffer, the sample was incubated in the MERSCOPE Gene Panel Mix at 37 °C for 42 h. Following probe hybridization, the sample was washed twice with the Formamide Wash Buffer at 47°C for 30 min and once with the Sample Prep Wash Buffer at RT for 2 min. After washing, the sample was embedded in hydrogel by incubating in the Gel Embedding Solution (Gel Embedding Premix (PN20300004), 10% w/v ammonium persulfate solution, and N, N, N’, N]′-tetramethylethylenediamine) at RT for 1.5 h. Then, the sample was cleared by first incubating in the Digestion Mix (Digestion Premix (PN 20300005) and 1:40 RNase inhibitor) at RT for 2 h, followed by incubation in Clearing Solution (Clearing Premix [PN 20300003] and Proteinase K) at 47°C for 24 h, then at 37 °C for 24 h. The cleared sample was washed twice with the Sample Prep Wash Buffer and stained with DAPI and PolyT Staining Reagent at RT for 15 min, then washed with the Formamide Wash Buffer at RT for 10 min and rinsed with the Sample Prep Wash Buffer. The sample was imaged with the MERSCOPE Instrument, and the detected transcripts were decoded on the same instrument using a Codebook generated by Vizgen. Transcripts were visualized on Vizgen’s Vizualizer.

### Single-nucleus RNA-seq pre-processing

Demultiplexed FASTQ files were used to generate gene-by-cell matrices with Cell Ranger (v6.0.0)^58^ and were aligned to the Arabidopsis nuclear transcriptome, prepared using the TAIR10 genome and the Araport 11 transcriptome. Downstream analyses were performed with the R^59^ package Seurat (v4.1.0)^60^. Low-quality and potential doublet nuclei that did not meet the cutoffs of >275 genes detected, <5,000 genes detected, >325 UMI per nucleus, <5% mitochondrial reads, and <10% chloroplast reads were removed from the datasets.

### Single-nucleus RNA-seq analysis

For the analysis of the 10X Genomics Gene Expression datasets, 10,000 variable features were used for data scaling and PCA calculation. Datasets were integrated by regressing ‘treatment’, ‘tissue type’, and ‘percent chloroplast read %’ using Harmony (v0.1.0)^61^. Dimensionality reduction, nearest neighbor identification, and clustering were performed using 20 harmony dimensions.

### Single-nucleus multiome pre-processing

Demultiplexed FASTQ files were used to generate gene-by-cell matrices and barcoded ATAC fragment files with Cell Ranger ARC (v2.0.2) and were aligned to the Arabidopsis nuclear transcriptome, as described previously, and the TAIR10 genome, respectively. Downstream analyses were performed with the R package Seurat (v5.0.0). Low-quality and potential doublet nuclei that did not meet a cutoff of > 500 and < 25,000 UMIs per nucleus and 1,000 > and < 70,000 ATAC fragment reads were removed from the datasets.

### Cell segmentation and spatial dataset generation

Cell segmentation was performed using Baysor v0.7.1^62^ on the processed transcript coordinate matrices with the following parameters: -s 6 -x global_x -y global_y -g gene -m 20 -p --n-clusters 10 --scale-std 90% --count-matrix-format ‘tsv’. The resulting spatial datasets were imported and analyzed with Seurat (v5.0.0), and spatial cells with fewer than 10 transcripts were removed. Meta-analysis of air-grown 3-day-old etiolated seedlings was also performed^56^.

### Spatial multi-modal integration

Single-nucleus transcriptomic, multiomic, and segmented spatial transcriptomic datasets were merged and normalized using SCTransform^63^ with the following parameters: residual.features=spatial_genes, return.only.var.genes=TRUE, clip.range = c(−10, 10). Principal component analysis was then performed on the 1,000 spatial targets, and datasets were integrated using Harmony^61^. Dimensionality reduction and clustering were then performed using the Harmony embeddings.

### Imputation

Imputation of the integrated multi-modal dataset was performed with ALRA^64^. The function ‘choose_k’ was performed on the integrated dataset, and the resulting value of k=41 was used for the function ‘RunALRA’ for imputation.

### ATAC analysis

Analysis of ATAC data was performed with Signac (v1.12.0)^65^. Briefly, ATAC blacklist regions defined by ^66^ were pre-filtered and reads mapping to organellar genomes were removed. Peak calling was performed on the ATAC data in bulk as well as on reads mapping to cell clusters of the multi-modal integrated dataset. Consensus peaks were identified by performing peak calling on each dataset independently using the CallPeaks command with the parameter ‘effective.genome.size = 119481543’, after which peaks were filtered for peakwidths of > 20bp and < 10,000bp. All identified peaks were concatenated, and overlapping peaks were merged to generate a set of consensus peaks. Quantification of chromatin accessibility within consensus peak regions was performed for all clusters. Cluster-specific peaks were identified by extracting fragment reads from cells in each cluster, performing *de novo* peak calling, and quantifying reads within peak regions.

### Motif analysis

Motif analysis of motifs within the JASPAR2020 database^67^ was performed on *de novo* identified peaks. Analysis of motif activity was performed using chromVAR (v1.20.2)^68^

### Gene regulatory network inference

Gene regulatory network construction and analysis of AHR cells was performed using scMEGA ^30^. The *BBM* motif, as described by^69^, was manually added to the list of motifs used for analysis. As binding motifs have not been previously determined for *GATA2* and *ARF6*, motifs for the nearest phylogenetic family members, *GATA9* and *ARF8,* were used as substitutions to correlate *GATA2* and *ARF6* promoter chromatin accessibility and expression with motif activity of putative target genes. The GRN was subsetted for TFs with motif activity and for target genes with a correlation > 0.4.

### Gibberellic acid treatment

Arabidopsis seeds were surface-sterilized as described previously and placed on 1X LS medium (Caisson) plates with 1 mg/ml PAC (APO456788397, Sigma Aldrich) + 10 nM GA3 (G7645, Sigma Aldrich) or on mock plates. The hormone solution contained 0.1% ethanol. A mock treatment control was also included. The seeds were stratified and germination was synchronized as described previously. Seedlings were grown in complete darkness with a control or ethylene gas flow for 3 days, as described previously, before imaging.

### Apical hook angle measurement

Apical hook angle measurement was performed as described in^11^. Angles between the hypocotyl and cotyledons were calculated with ImageJ^70^ using the ‘ObjectJ’ plugin^71^. For the statistical analysis, an unpaired t-test was performed on the apical hook angle of *gata2* mutants vs WT seedlings for each experimental condition.

## Supplementary Material

Supplement 1 Extended Data Fig. 1. Identification and annotation of cell types in the integrated Arabidopsis seedling dataset.
Extended Data Fig. 2. Clusters and cell types respond heterogeneously to ethylene treatment.
Extended Data Fig. 3. Single-nucleus RNA-seq captures transient ethylene-induced ectopic root hair development.
Extended Data Fig. 4. Cells of the seedling apex represent diverse cell types and cell states.
Extended Data Fig. 5. Single-cell analysis of segmented spatial datasets identifies seedling cell types.
Extended Data Fig. 6. Integration of spatial datasets identifies clusters that correspond to cell types and cell states.
Extended Data Fig. 7. Spatial multi-modal integration reveals spatially distinct cell type subpopulations.
Extended Data Fig. 8. Asymmetric accumulation of developmental regulators is specific to cells within the apical hook.
Extended Data Fig. 9. Cell state markers functionally regulate apical hook angle and exaggeration in air and ethylene treated conditions.
Extended Data Fig. 10. Cell state markers functionally regulate apical hook angle and exaggeration within AHR cells.
Extended Data Fig. 11. Cluster 26 cells correspond to the AHR.
Extended Data Fig. 12. Divergent regulation of GA metabolism enzymes functionally regulate cell elongation asymmetrically within AHR cell subpopulations.
Extended Data Fig. 13. Divergent gene regulatory networks functionally regulate cell elongation asymmetrically within ARH cells.
Extended Data Fig. 14. Asymmetric accumulation of GRN targets associated with cell elongation suppression within identical files of AHR cells.
Extended Data Fig. 15. Exogenous GA treatment does not fully rescue the hyper exaggerated apical hook phenotype of seedlings with dual ethylene + GA treatment.

Supplement 2 Supplementary Table 1. Cluster markers identified from the integrated seedling dataset.

Supplement 3 Supplementary Table 2. Genes upregulated by ethylene treatment within individual seedling clusters.

Supplement 4 Supplementary Table 3. Genes downregulated by ethylene treatment within individual seedling clusters.

Supplement 5 Supplementary Table 4. Cluster markers identified from the re-clustered apical seedling dataset.

Supplement 6 Supplementary Table 5. Cluster markers identified from the multi-modal spatial integrated dataset.

Supplement 7 Supplementary Table 6. Cluster specific peaks and enriched motifs identified from the multi-modal spatial integrated dataset.

Supplement 8 Supplementary Table 7. Subcluster specific markers and enriched motifs within accessible chromatin regions identified from the apical hypocotyl cell re-clustering (cluster 26).

Supplement 9 Supplementary Table 8. Full and subset gene regulatory networks.

## Data Availability

Raw and processed datasets have been deposited at Gene Expression Omnibus (accession numbers GSE326975, GSE327163, and GSE327709. Interactive access to the single-cell datasets is available at http://arabidopsisdevatlas.salk.edu/. Code used in this study can be found at https://github.com/travandlee/ethylene_atlas.
