# Supplementary material for: Transient cell states encode positional information to direct asymmetric growth
Source: bioRxiv. 2026 May 22:2026.05.21.726925. Preprint. [Version 1] doi: 10.64898/2026.05.21.726925 (PMC13228422; doi:10.64898/2026.05.21.726925)
Supplement: Supplement 1 — Extended Data Fig. 1. Identification and annotation of cell types in the integrated Arabidopsis seedling dataset. Extended Data Fig. 2. Clusters and cell types respond heterogeneously to ethylene treatment. Extended Data Fig. 3. Single-nucleus RNA-seq captures transient ethylene-induced ectopic root hair development. Extended Data Fig. 4. Cells of the seedling apex represent diverse cell types and cell states. Extended Data Fig. 5. Single-cell analysis of segmented spatial datasets identifies seedling cell types. Extended Data Fig. 6. Integration of spatial datasets identifies clusters that correspond to cell types and cell states. Extended Data Fig. 7. Spatial multi-modal integration reveals spatially distinct cell type subpopulations. Extended Data Fig. 8. Asymmetric accumulation of developmental regulators is specific to cells within the apical hook. Extended Data Fig. 9. Cell state markers functionally regulate apical hook angle and exaggeration in air and ethylene treated conditions. Extended Data Fig. 10. Cell state markers functionally regulate apical hook angle and exaggeration within AHR cells. Extended Data Fig. 11. Cluster 26 cells correspond to the AHR. Extended Data Fig. 12. Divergent regulation of GA metabolism enzymes functionally regulate cell elongation asymmetrically within AHR cell subpopulations. Extended Data Fig. 13. Divergent gene regulatory networks functionally regulate cell elongation asymmetrically within ARH cells. Extended Data Fig. 14. Asymmetric accumulation of GRN targets associated with cell elongation suppression within identical files of AHR cells. Extended Data Fig. 15. Exogenous GA treatment does not fully rescue the hyper exaggerated apical hook phenotype of seedlings with dual ethylene + GA treatment. [file media-1.pdf]

Supplementary Materials for

Transient cell states encode positional information to direct asymmetric growth

Authors: Travis A. Lee[1,2], Kamonkan Gamnerdsiri[1], Natanella Illouz-Eliaz[1], Tatsuya Nobori[1]†, Joseph R. Nery[3], Bruce Jow[3], Michelle Liem[4], Caz O'Connor[4], Joseph R. Ecker[2,3]*

Corresponding author: ecker@salk.edu

The PDF file includes:

Extended Data Figs. 1 to 15

Other Supplementary Materials for this manuscript include the following:

Supplementary Tables 1 to 8

Supplemental figures

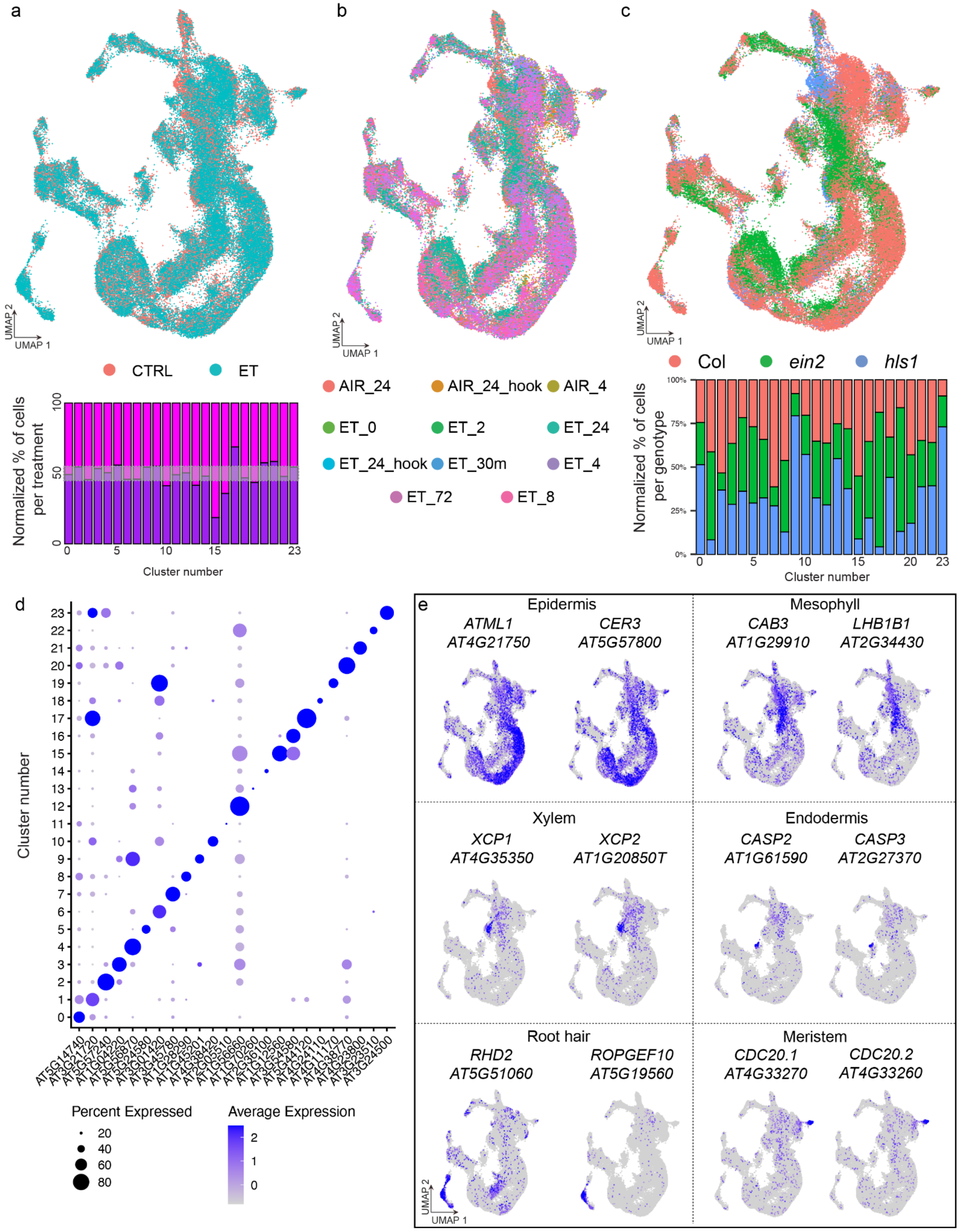

Extended Data Fig. 1. Identification and annotation of cell types in the integrated Arabidopsis seedling dataset.
a-c, Depiction (top) and quantitation (bottom) of single-nucleus RNA-seq datasets that correspond to conditions (**a**), treatments (**b**), and genotypes (**c**) of plants included in the integrated seedling dataset. Nuclei correspond to the dataset, treatment, or genotype as indicated. **d,** Top markers identified for each cluster. **e,** Expression of known cell type markers within the integrated seedling dataset that correspond to epidermis, mesophyll, xylem, endodermis, root hair, and meristem cell types.

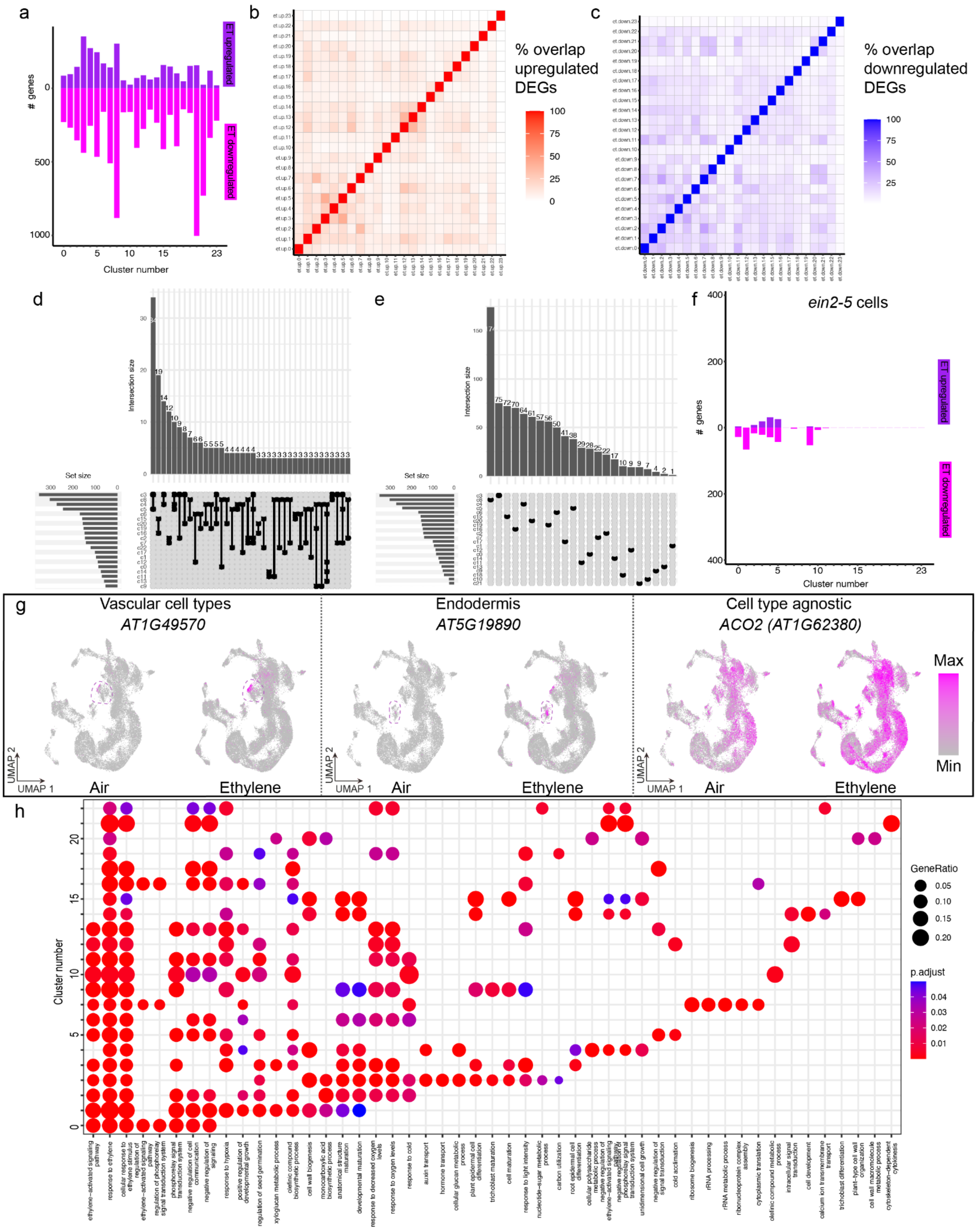
a
ET upregulated
ET downregulated
genes
Cluster number
b
% overlap upregulated DEGs
c
% overlap downregulated DEGs
d
e
Intersection size
Set size
f
ein2-5 cells
g
Vascular cell types
AT1G49570
Endodermis
AT5G19890
Cell type agnostic
ACO2 (AT1G62380)
Max
Min
UMAP 1
UMAP 2
Air
Ethylene
h
Cluster number
GeneRatio
p.adjust
ethylene-activated signaling pathway
response to ethylene
cellular response to ethylene stimulus
response to hypoxia
response to decreased oxygen levels
response to oxygen levels
response to cold
cell wall biogenesis
auxin transport
hormone transport
ribosome biogenesis
rRNA processing
cytoplasmic translation
cell development
cytoskeleton-dependent cytokinesis

Extended Data Fig. 2. Clusters and cell types respond heterogeneously to ethylene treatment

a, Number of differentially expressed (DE) genes identified between control and ethylene-treated conditions of cells within each cluster. FDR < 0.05. **b,c,** Percent overlap of genes upregulated (**b**) or downregulated (**c**) by ethylene treatment between all clusters of the seedling dataset. **d,e,** Upset plots of ethylene upregulated genes identified among clusters. Ethylene upregulated genes shared between two or more clusters (**d**, greater than two per category group) or ethylene-induced genes unique to single clusters (**e**) are depicted. **f,** Number of DE genes between control and ethylene-treated conditions of only *ein2-5* cells. FDR < 0.05. **g,** Representative examples of cell type-specific (vascular and endodermis cell types) and cell type-agnostic ethylene-induced genes. **h,** GO term enrichment of ethylene-induced genes identified for each cluster.

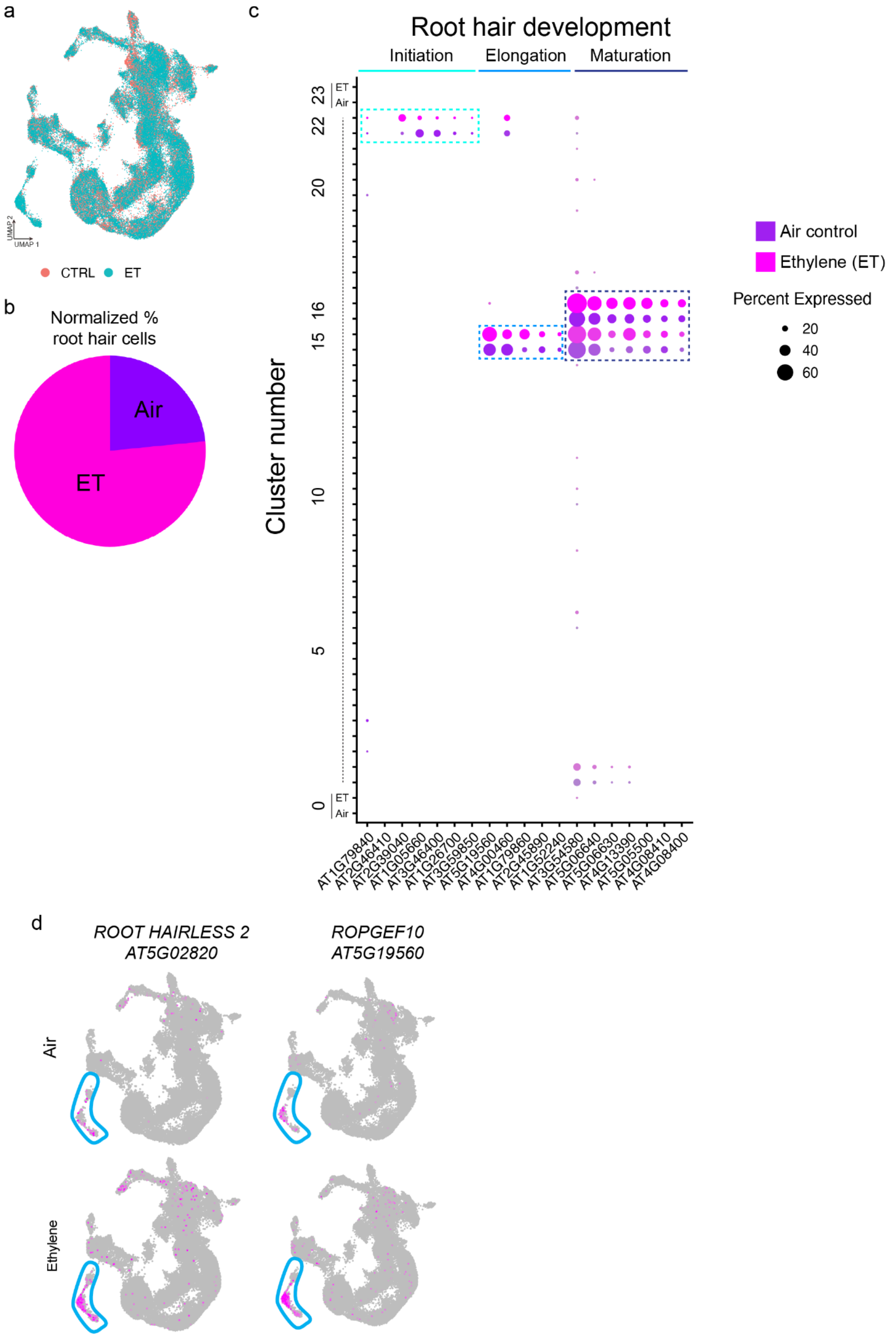

Extended Data Fig. 3. Single-nucleus RNA-seq captures transient ethylene-induced ectopic root hair development.

a,b, Root hair cell clusters (clusters 15, 16, and 22) (**a**) are overrepresented in ethylene-treated conditions compared to control (**b**). **c,** Split dotplot expression of genes involved in root hair initiation, elongation, and maturation. For each cluster, expression is depicted for control and treated cells, which are depicted in purple and magenta, respectively. **d,** Representative images of select root hair development genes are depicted in control and ethylene-treated cells.

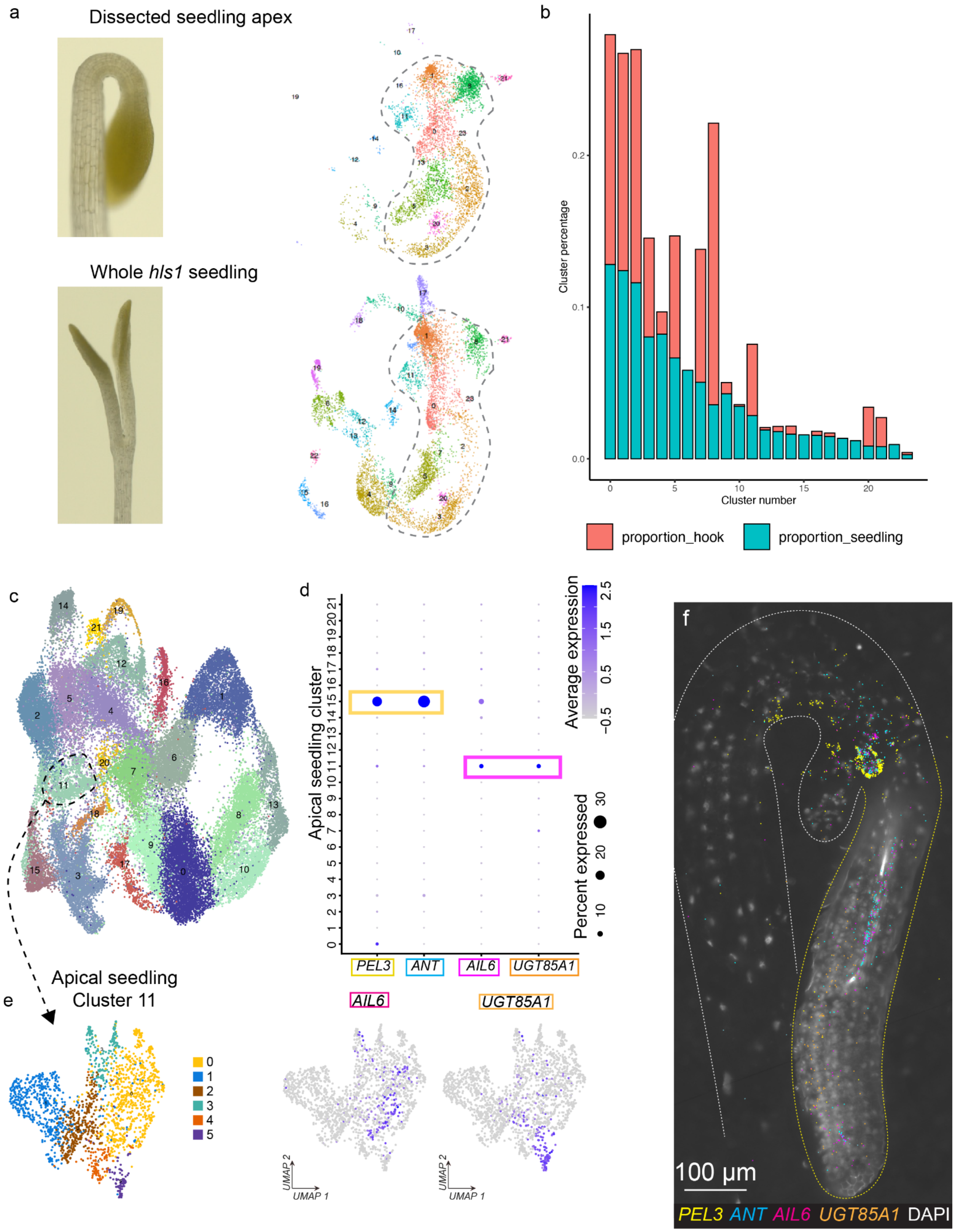

Extended Data Fig. 4. Cells of the seedling apex represent diverse cell types and cell states. **a,** Cells in the dissected seedling apex and *hls1* whole seedlings uniquely represent and are depleted for the same clusters. **b,** Quantitation of cells corresponding to whole seedling samples

and dissected apical hook samples. **c,** Re-clustering of the nine clusters that represent the apical hook and shoot apex of seedlings. **d,** Expression of select marker genes that correspond to young cotyledon (cluster 15; *PEL3* [*PERMEABLE LEAVES3*; *AT5G23940*], ANT [*AINTEGUMENTA*; *AT4G37750*]) and meristematic (cluster 11; *AIL6* [*AINTEGUMENTA-LIKE 6*; *AT5G10510*] and *UGT85A1* [*AT1G22400*]) cell clusters. **e,** Re-clustering of the meristematic cell cluster (cluster 11) with expression of the cluster 11 markers *AIL6* and *UGT85A1* within the re-clustered cells. **f,** Representative single-molecule detection of *PEL3*, *ANT*, *AIL6*, and *UGT85A1* in the seedling apical hook and shoot apex region. Scale bar, 100µm.

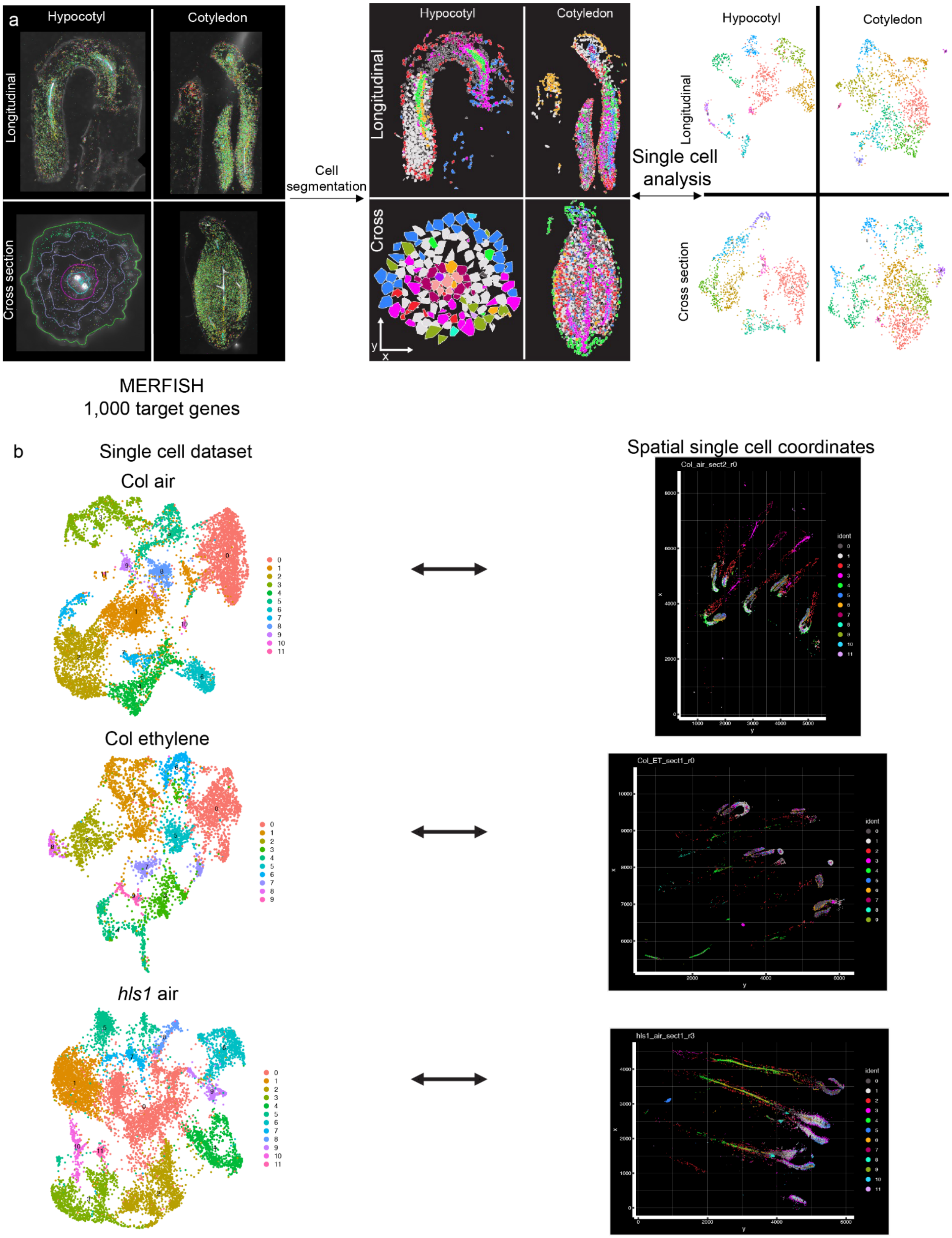

Extended Data Fig. 5. Single-cell analysis of segmented spatial datasets identifies seedling

cell types.
a, Diagram of analyses performed for single-molecule MERFISH datasets, including cell segmentation and standard single-cell analyses. **b,** Representative clustering results and spatial coordinates of MERFISH single-cell datasets. Cells are colored according to cluster identity, as depicted in the corresponding UMAP.

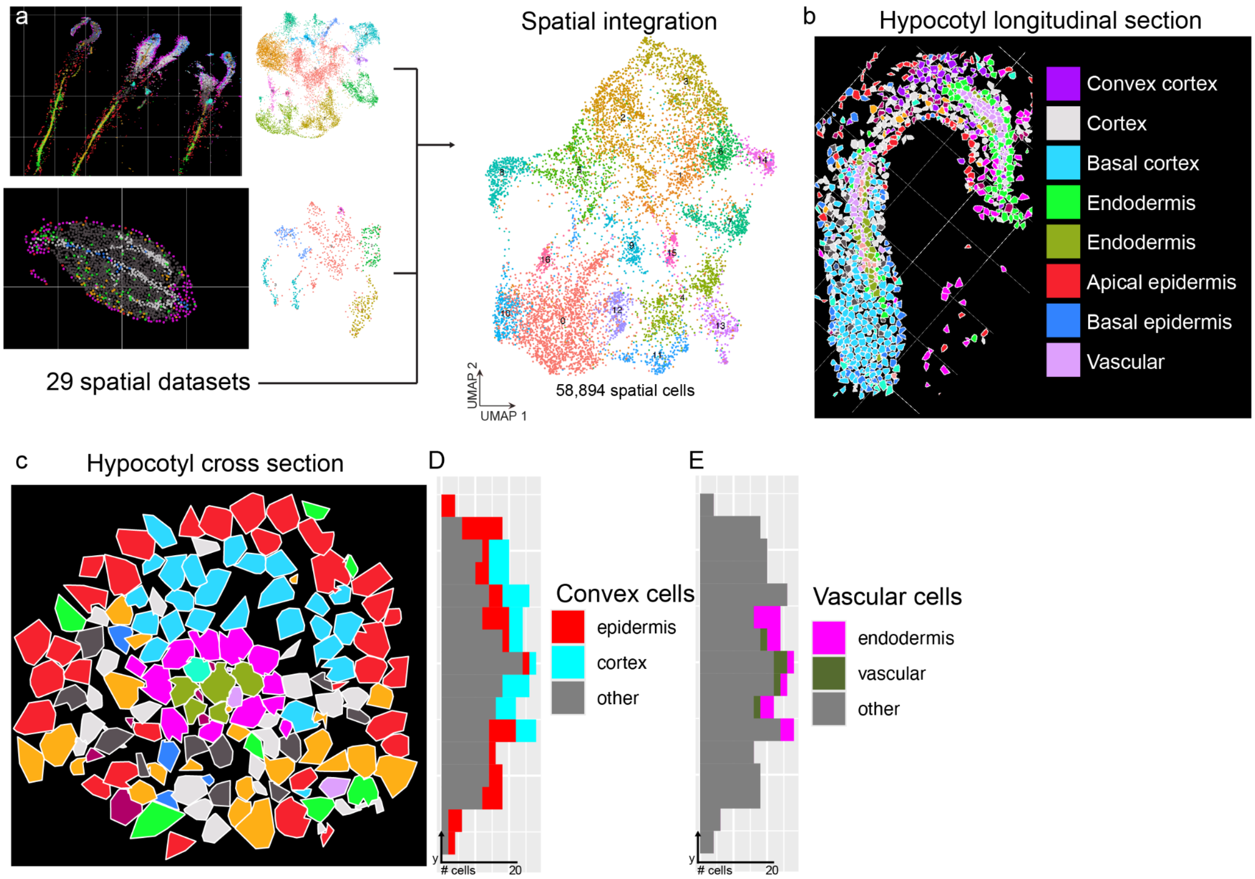

Extended Data Fig. 6. Integration of spatial datasets identifies clusters that correspond to cell types and cell states.

a, Diagram of integration of all spatial datasets and the resulting dimensionality reduction. **b,c,** Spatial cell coordinates with annotated clusters of a longitudinal (**b**) and cross-section (**c**) of the apical hook section (corresponds to Fig. 2B). **d,e,** Quantitation of epidermis and cortex cell types (**d**) and endodermis and vascular cell types (**e**) across spatial bins along the y-axis.

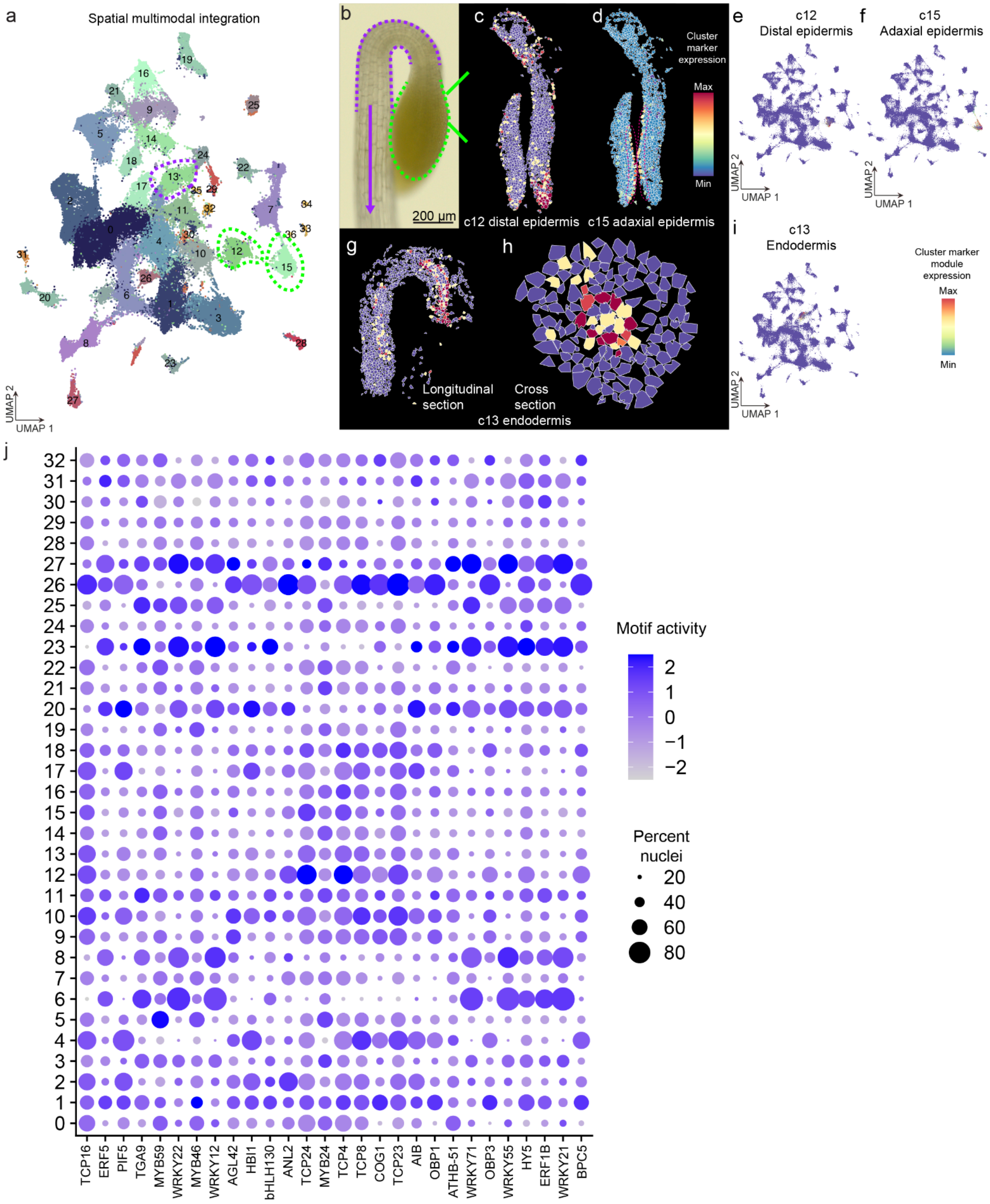

Extended Data Fig. 7. Spatial multi-modal integration reveals spatially distinct cell type subpopulations.

a, Spatial multi-modal integration of single-nucleus RNA and multiomic datasets with spatial

cell datasets. Clusters corresponding to distal epidermal (cluster 12) and adaxial epidermal (cluster 15) cell populations are circled for emphasis. **b,** Representative image of regions within the apical hook. **c,d,** Spatial expression of distal epidermis (**c**) and adaxial epidermis (**d**) markers within cotyledons and the AHR. **e,f,** Expression of distal epidermis and adaxial epidermis markers in the spatial multi-modal dataset. **g,h** Spatial expression of endodermis markers (cluster 13) in longitudinal (**g**) and cross sections (**h**) of hypocotyls. **i,** Expression of endodermis markers in the spatial multi-modal dataset. **j,** Top motif within accessible chromatin (motif activity) for each cluster.

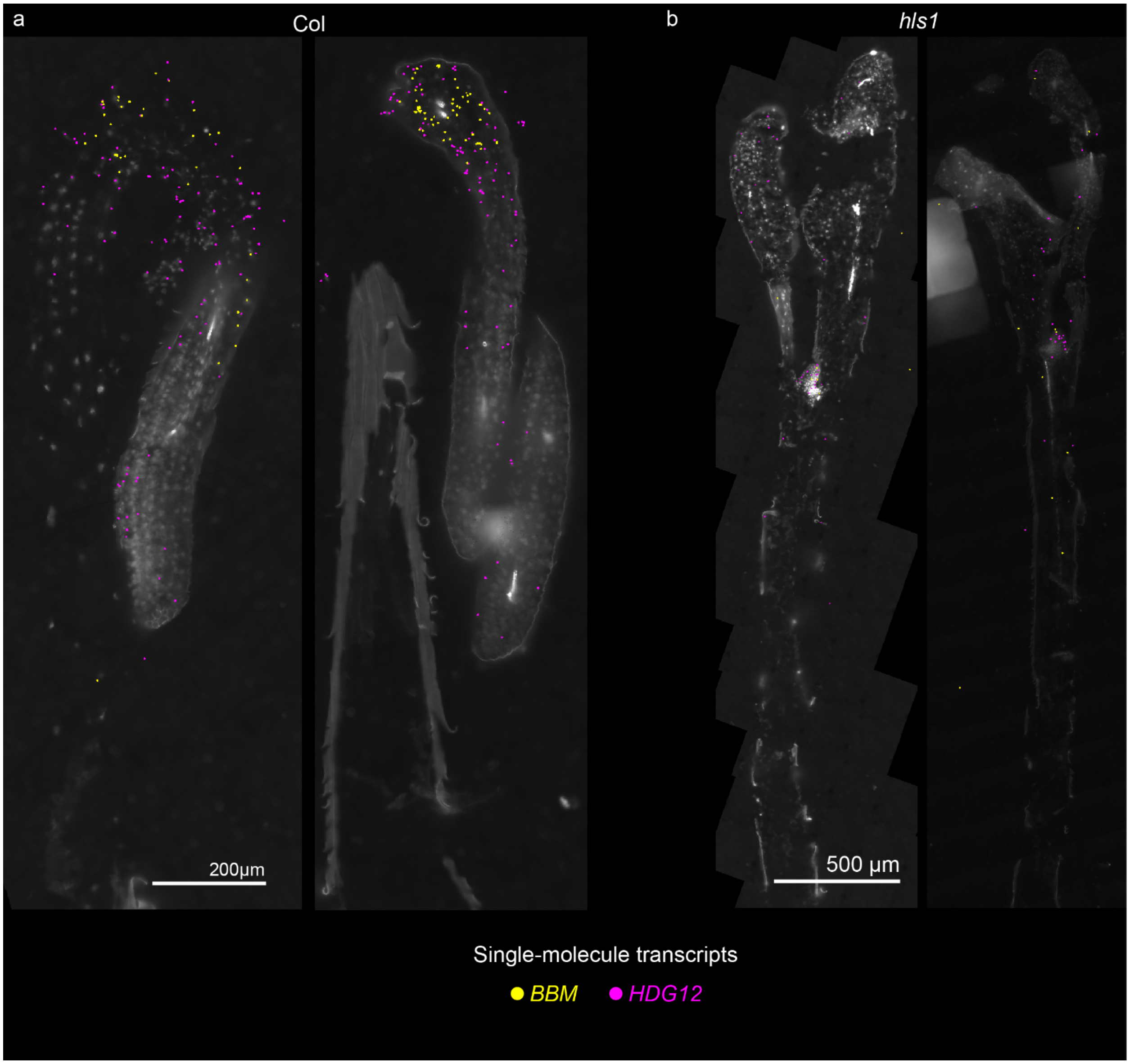

Extended Data Fig. 8. Asymmetric accumulation of developmental regulators is specific to cells within the apical hook.

a,b, Spatial single-molecule detection of *BBM* (yellow) and *HDG12* (magenta) in the apical hook of WT (**a**) and straightened hypocotyl of *hls1* seedlings (**b**). Scale bars, 200µm and 500µm, respectively.

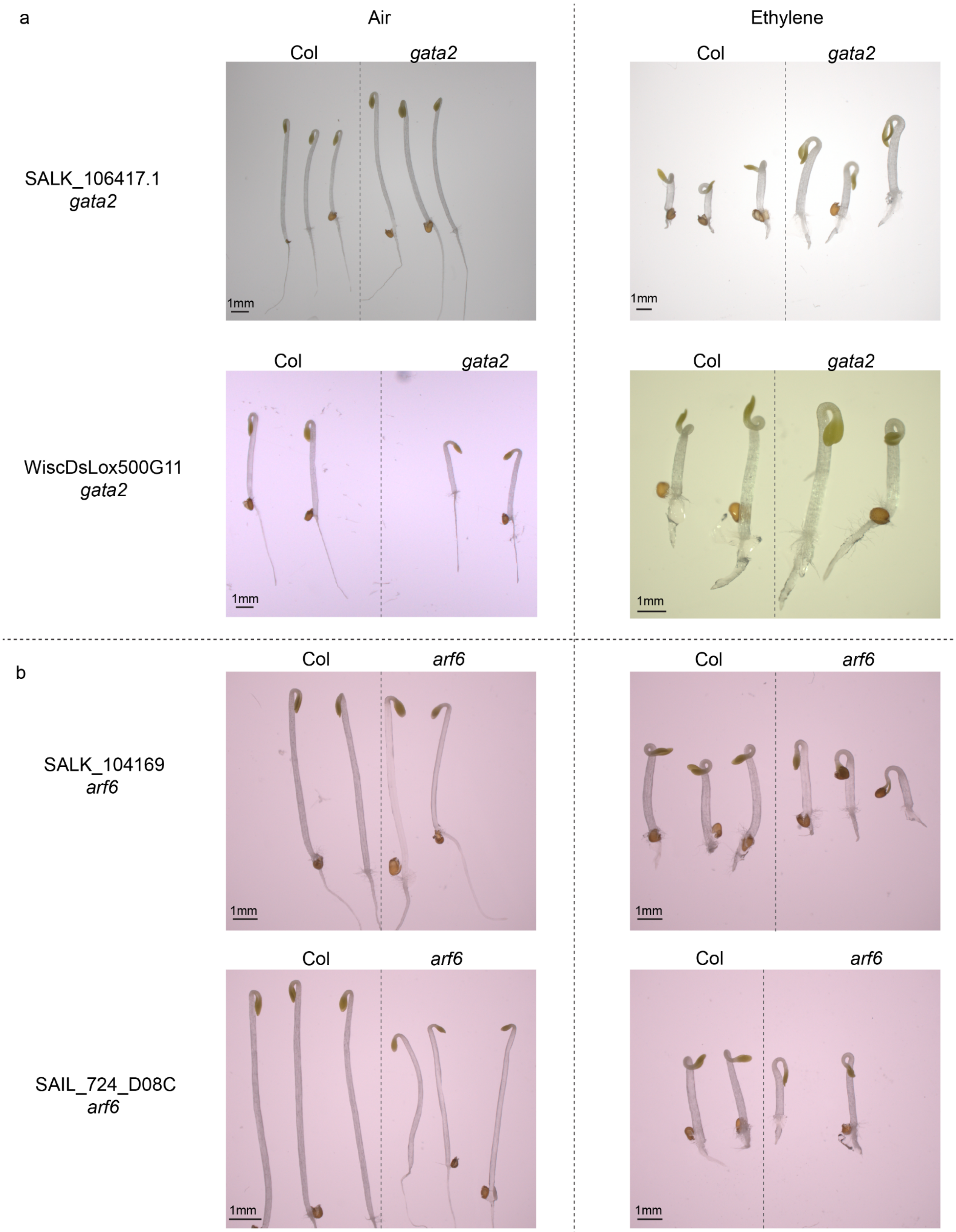

Extended Data Fig. 9. Cell state markers functionally regulate apical hook angle and exaggeration in air and ethylene treated conditions

a,b, Apical hook angle and apical hook exaggeration are perturbed in *gata2* (**a**) and *arf6* mutants

(**b**) when grown in control (air) and ethylene treated conditions, respectively. Scale bar, 1mm.

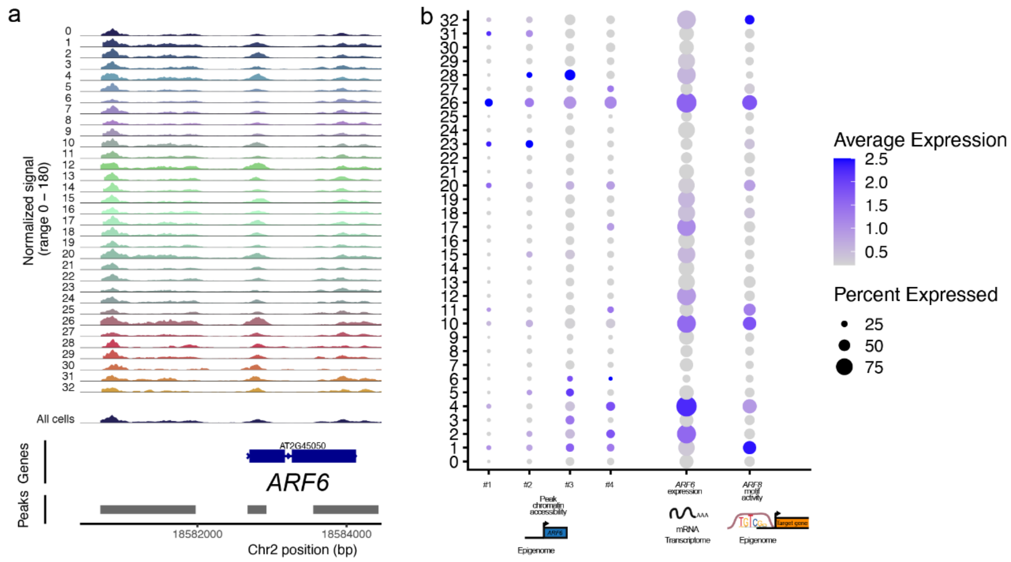

Extended Data Fig. 10. Cell state markers functionally regulate apical hook angle and exaggeration within AHR cells.

a, Chromatin accessibility within the ARF6 promoter. **b,** Quantification of chromatin accessibility within peaks in the *ARF6* promoter, expression of *ARF6*, and motif activity of putative *ARF6* target genes.

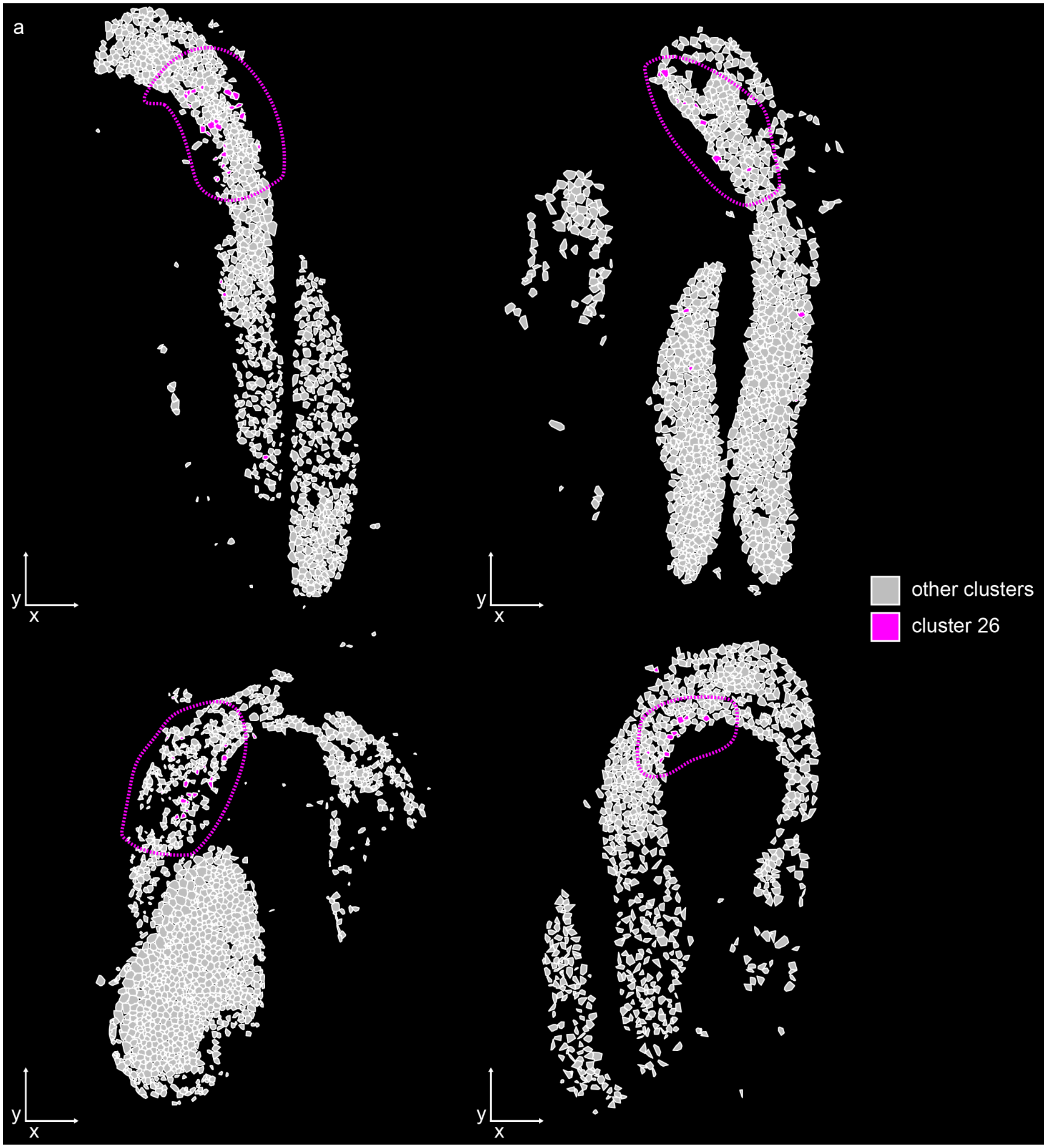

Extended Data Fig. 11. Cluster 26 cells correspond to the AHR
a, Spatial identification of cluster 26 cells in the AHR of various seedlings. Cluster 26 cells are colored pink, all other cells are colored gray. Apical hook regions that contain cluster 26 cells are circled for emphasis.

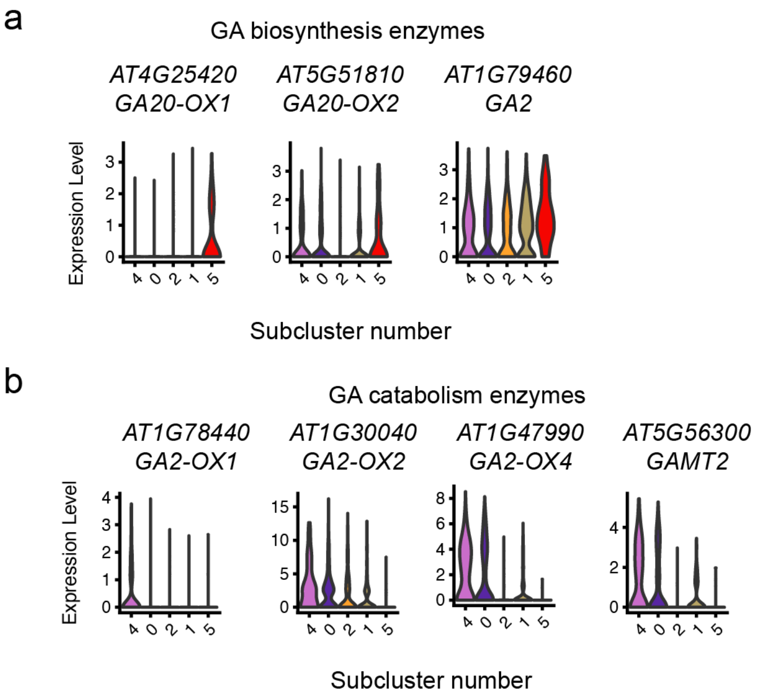

Extended Data Fig. 12. Divergent regulation of GA metabolism enzymes functionally regulate cell elongation asymmetrically within AHR cell subpopulations.
a,b, Expression of GA biosynthesis (**a**) and GA catabolism (**b**) genes within the AHR re-clustered cells.

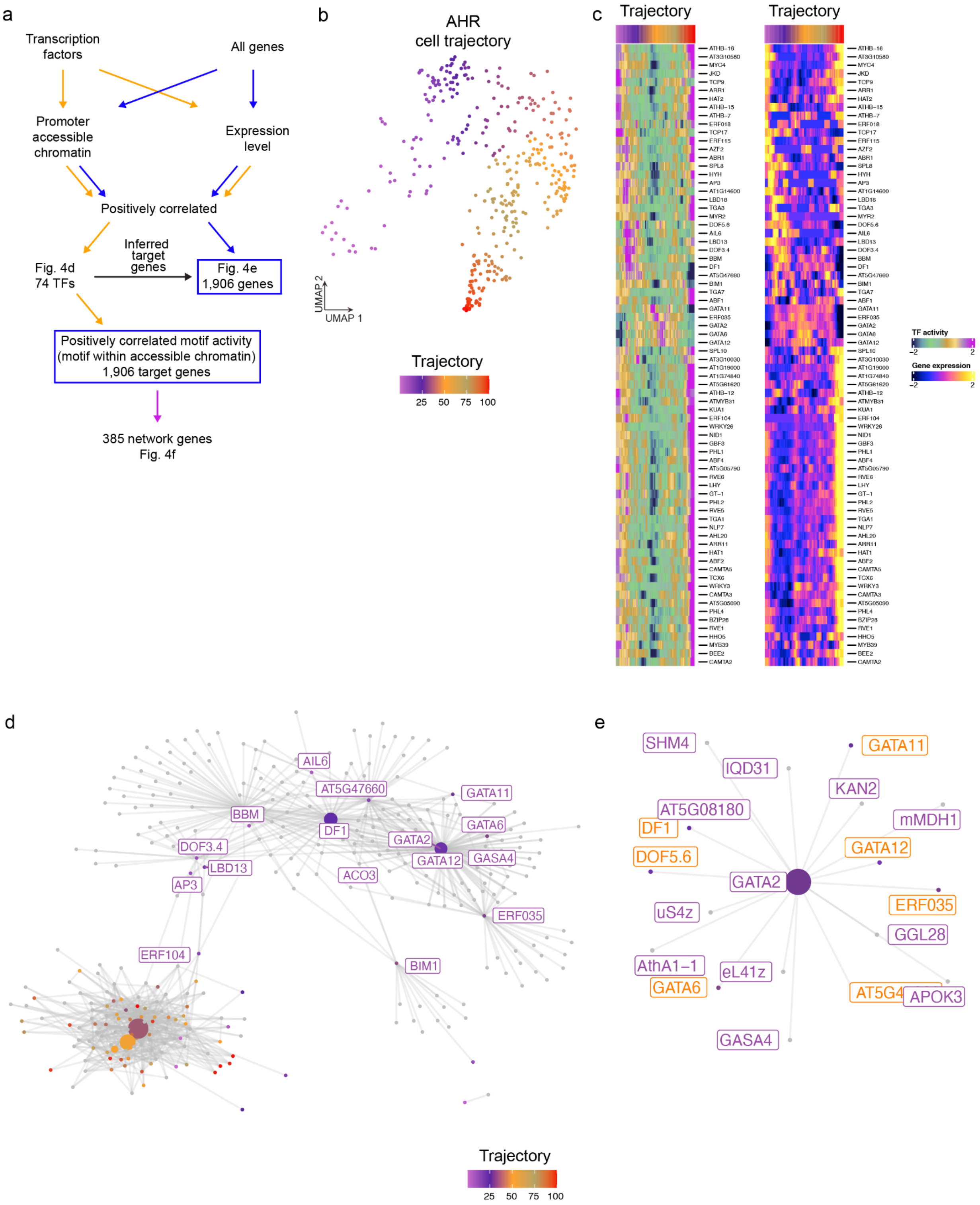

Extended Data Fig. 13. Divergent gene regulatory networks functionally regulate cell elongation asymmetrically within ARH cells.

a, Flow diagram of gene regulatory network nodes and target identification. **b,** Calculated cell trajectory of AHR re-clustered cells. **c,** Fully annotated heatmap of TF expression and motif activity of target genes. **d,** Gene regulatory network plot of the filtered GRN presented in Fig.

4F. Transcription factor nodes are colored based on their expression within the cell trajectory. Select TF nodes are labeled. **e,** Gene regulatory network plot of predicted *GATA2* targets. A full list of all TF-node interactions is presented in Supplemental Table 8.

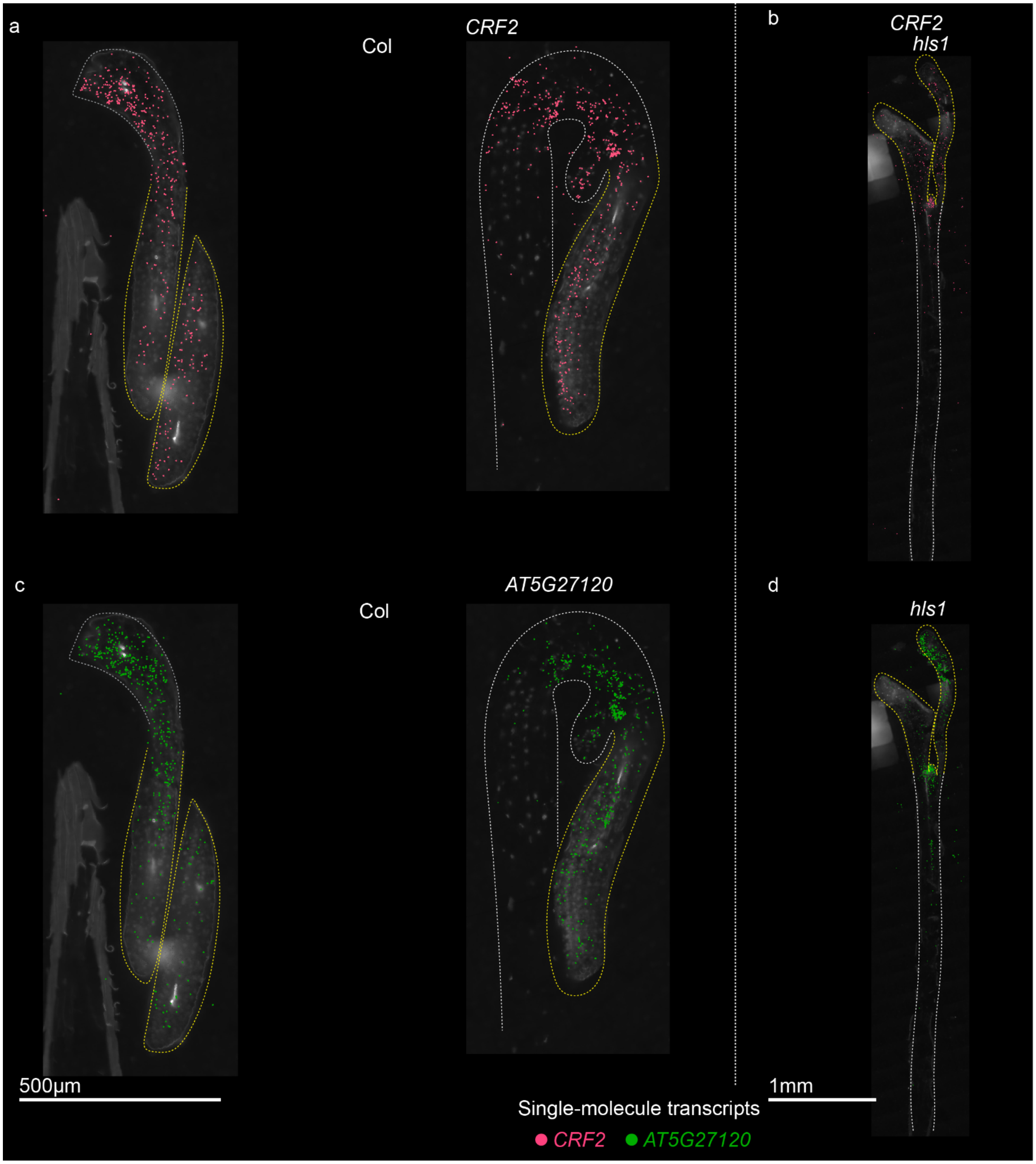

Extended Data Fig. 14. Asymmetric accumulation of GRN targets associated with cell elongation suppression within identical files of AHR cells.
a,b, Single-molecule detection of *CRF2* transcript in the AHR of WT seedlings (**a**) and *hls1* seedlings (**b**). **c,d,** Single-molecule detection of *AT5G27120* transcript in the AHR of WT seedlings (**c**) and *hls1* seedlings (**d**).

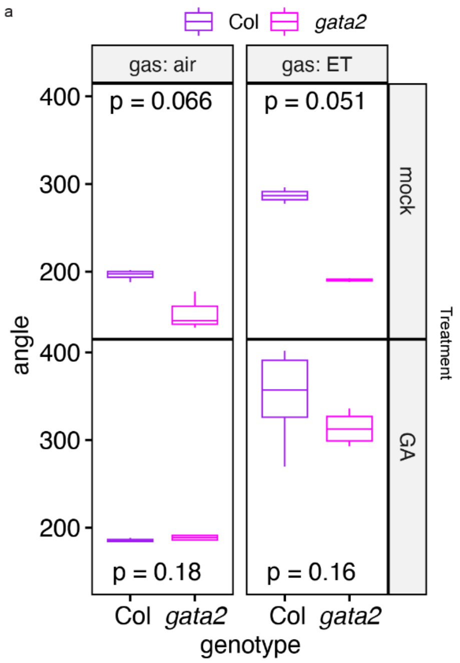

Extended Data Fig. 15. Exogenous GA treatment does not fully rescue the hyper exaggerated apical hook phenotype of seedlings with dual ethylene + GA treatment.
a, Quantitation of apical hook angle of Col and *gata2* mutants in air or ethylene-treated conditions, when grown on mock or PAC + GA treated media. Apical hook angle is displayed as box plots; the centre line indicates the median value, the box boundaries represent the 25th and 75th percentiles, and the whiskers extend to the minimum and maximum values within 1.5 times the interquartile range. p-values determined by unpaired t-test comparing the mutant to WT for each treatment combination is depicted. Apical hook angle was measured for n = 4 seedlings for all combinations of genotypes and treatments aside from *gata2* grown in mock air conditions (n = 3) and Col and *gata2* grown in mock ethylene conditions (n = 2).

Supplemental Data

Supplemental Table 1. Cluster markers identified from the integrated seedling dataset

Supplemental Table 2. Genes upregulated by ethylene treatment within individual seedling clusters

Supplemental Table 3. Genes downregulated by ethylene treatment within individual seedling clusters

Supplemental Table 4. Cluster markers identified from the re-clustered apical seedling dataset

Supplemental Table 5. Cluster markers identified from the multi-modal spatial integrated dataset

Supplemental Table 6. Cluster-specific peaks and enriched motifs identified from the multi-modal spatial integrated dataset

Supplemental Table 7. Subcluster-specific markers and enriched motifs within accessible chromatin regions identified from the apical hypocotyl cell re-clustering (cluster 26)

Supplemental Table 8. Full and subset gene regulatory network
